# Routine screening of abnormal vaginal flora during pregnancy reduces the odds of preterm birth: a systematic review and meta-analysis

**DOI:** 10.1038/s41598-023-40993-x

**Published:** 2023-08-25

**Authors:** Eszter Hoffmann, Szilárd Váncsa, Alex Váradi, Péter Hegyi, Rita Nagy, Balázs Hamar, Vanda Futács, Begüm Kepkep, Péter Nyirády, Csaba Demendi, Nándor Ács

**Affiliations:** 1 Department of Obstetrics and Gynecology, Semmelweis University, Budapest, Üllői út, 78/A, 1082 Budapest, Hungary; 2 Centre for Translational Medicine, Semmelweis University, Budapest, Hungary; 3 Institute for Translational Medicine, Medical School, University of Pécs, Pécs, Hungary; 4 Division of Pancreatic Diseases, Heart and Vascular Center, Semmelweis University, Budapest, Hungary; 5 Heim Pál National Pediatric Institute, Budapest, Hungary; 6 Department of Urology, Semmelweis University, Budapest, Hungary

**Keywords:** Disease prevention, Health care economics, Public health, Epidemiology, Translational research

## Abstract

Prematurity is the leading cause of perinatal mortality and the morbidity among children under the age of 5. The prevalence of preterm birth is between 5 and 18% worldwide. Approximately 30% of preterm deliveries occur as a consequence of fetal or maternal infections. Bacterial vaginosis can increase the risk of ascending infections. However, there is no recommendation or protocol for screening of abnormal vaginal flora. The aim of this systematic review was to investigate the effectiveness of routine screening of abnormal vaginal flora during pregnancy care. We conducted our systematic search in the following databases: MEDLINE via PubMed, Embase, and Cochrane Library. Studies reporting on pregnant women with no symptoms of bacterial vaginosis were included in our analysis if they provided data on the outcome of their pregnancy. The intervention group went through screening of abnormal vaginal flora in addition to routine pregnancy care. Odds ratio (OR) with 95% confidence intervals (CIs) was used as effect size measure. From each study the total number of patients and number of events was extracted in both the intervention and control arm to calculate OR. Altogether we included 13 trials with 143,534 patients. The screening methods were Gram stain, pH screening, pH self-screening and pH screening combined with Gram stain. Regular screening of vaginal flora compared to no screening significantly reduces the odds of preterm birth before 37 weeks (8.98% vs 9.42%; OR 0.71, CI 0.57–0.87), birthweight under 2500 g (6.53% vs 7.24%; OR 0.64, CI 0.50–0.81), preterm birth before 32 weeks (1.35% vs 2.03%; OR 0.51, CI 0.31–0.85) and birthweight under 1000 g (0.86% vs 2.2%; OR 0.33, CI 0.19–0.57). In conclusion, the routine screening of abnormal vaginal flora might prevent preterm birth, extreme preterm birth, low birthweight deliveries and very low birthweight deliveries. Further research is needed to assess the problem more accurately.

## Introduction

Prematurity is the leading cause of perinatal mortality and morbidity in the developed world. The prevalence of preterm birth is between 5 and 18% worldwide, and despite intensive efforts and clinical advancement, the rate of preterm births in industrialized countries has not changed or has even increased^1–3^. This equals 15 million premature infants annually^3^. The reduction of preterm birth rates is considered a public health priority because of its short-term and long-term consequences^4^.

Preterm delivery can be spontaneous or provider-initiated (cesarean section or labor induction) because of fetal or maternal indications. Spontaneous preterm delivery is associated with various epidemiological and clinical risk factors, with intrauterine infection being one of its most prevalent^1,5^. Up to 40% of spontaneous preterm deliveries occur because of intrauterineinfections, usually asymptomatic^6^.

Bacteria causing intrauterine infections can access the chorioamnion either by ascending from the vagina or through the placenta as hematogenous dissemination of various infections such as urinary tract infection, pneumonia, periodontal disease, or appendicitis^7^. The most common pathway of intrauterine infections is the ascending route^1^. Therefore, bacterial vaginosis is generally considered a risk factor for preterm delivery^8,9^. Despite all this data, there are no recommendations or protocols for routine screening of abnormal vaginal flora, even though there are easily executable screening methods available, such as Gram stain, pH screening, pH self-screening, and the combination of these techniques.

The role of routine screening of abnormal vaginal flora during pregnancy in preventing preterm births is controversial. On the one hand, in several European studies, abnormal vaginal flora screening seemed to prevent preterm deliveries successfully^10–12^. On the other hand, the recently published American College of Obstetricians and Gynecologists (ACOG) Practice Bulletin declares insufficient data to recommend vaginal flora screening as a routine practice^13^. This recommendation states with moderate certainty that screening for asymptomatic bacterial vaginosis in pregnant women with no increased risk for preterm delivery has no net benefit in preventing preterm delivery. It also states that the current evidence was insufficient to assess the benefits of screening for bacterial vaginosis in pregnant persons at increased risk for preterm delivery^14–16^.

This study aims to synthesize evidence-based data investigating the effectiveness of routine abnormal vaginal flora screening in preventing preterm delivery. We hypothesized that screening with any available tools could help decrease the rate of preterm deliveries.

## Methods

We report our systematic review and meta-analysis based on the recommendation of the PRISMA 2020 guideline^17^ (Table S1), while we followed the Cochrane Handbook^18^. The study protocol was registered on PROSPERO (CRD42021283212). Our minimum criteria for conducting a meta-analysis was to include at least three studies in each outcome. However, we performed a subgroup analysis of fewer than three studies and included these results in the qualitative synthesis.

### Eligibility criteria

Our clinical question was based on the PICO framework. Studies reporting on pregnant women without symptoms of vaginal infection (P) were included in our analysis if they provided data on the outcome of the pregnancies. In the intervention group (I), general screening of abnormal vaginal flora was performed in addition to routine pregnancy care, which also included the treatment of abnormal vaginal flora if diagnosed. The control group (C) participants underwent routine pregnancy care without screening the vaginal flora or a pre-defined protocol for screening. The primary outcome (O) was preterm delivery before 37 weeks. Data were also provided for the following secondary outcomes: preterm delivery before 34 and 32 weeks and birthweight < 2500 g, < 2000 g, < 1500 g, and < 1000 g.

For the intervention group, we did not have a pre-defined method for screening. Gram stain, pH-self screening, pH-screening provided by healthcare personnel, or the combination of these protocols were also acceptable. On the other hand, we did not have an exclusion criterion based on the frequency, number of screenings, or treatment of the abnormal vaginal flora. The first preference was the inclusion of randomized controlled trials (RCTs). However, we included prospective and retrospective observational trials due to the low number of RCTs. Conference abstracts were excluded from our study.

In the case of an overlapping population for a given outcome, we included the study with a larger sample size in our analysis.

### Information sources and search strategy

Our systematic search was conducted on the 21st of October 2021. We searched in the following databases: MEDLINE via PubMed, Embase, and Cochrane Library.

During the systematic search, we used a pre-defined search key *((pregnant OR pregnancy) AND (‘abnormal vaginal flora’ OR ‘vaginal pH’ OR ‘vaginal microbiota’ OR ‘vaginal infection’ OR ‘vaginal discharge’ OR ‘bacterial vaginosis’) AND (screening OR test)).* No language, study type, or other filters were applied.

### Selection process

Two independent review authors performed the selection after duplicate removal. First, we continued the selection by title and abstract, and then we excluded the inappropriate articles based on full text. On every level of the selection, a third independent review author resolved disagreements.

In the case of two trials, the population included multiple pregnancies^19,20^. However, the ratios of multiple pregnancies in the intervention and control groups were not different. Therefore, we decided to include these two papers in our meta-analysis based on these data.

### Data collection process and data items

From the eligible articles, two authors collected data independently. We created a standardized data collection sheet based on the consensus of methodological and clinical experts. A third independent reviewer resolved disagreements.

The following data were extracted: first author, the year of publication, study population, study period, study type, demographic data about the population, details of the interventions, event numbers, and the total number of patients in both intervention and control groups.

### Study risk of bias assessment and evidence level

For randomized controlled trials, we used the Cochrane risk-of-bias tool for randomized trials (RoB 2)^21^, while for non-randomized interventional trials, we used the Risk Of Bias In Non-Randomized Studies-of Interventions (ROBINS-I)^22^. Two independent review authors did the assessment, and a third independent investigator resolved the disagreements.

We followed the recommendation of the Grades of Recommendation, Assessment, Development, and Evaluation (GRADE) workgroup to evaluate the quality of evidence^23^.

### Synthesis methods

All statistical analyses were made with R (R Core Team 2020, v4.0.3) using the *meta* (v5.2.0)^24^ and *dmetar* (v0.0.9)^25^ packages.

The random-effects model was used to calculate odds ratio (OR) with a 95% confidence interval (CI). Forest plots were used to graphically summarize the results irrespective of the number of studies included in the pooled analysis. Forest plots with less than three studies were interpreted with limitations.

Where applicable, we reported the prediction intervals (i.e., the expected range of effects of future studies) of results following the recommendations of IntHout et al.^26^.

Cochrane’s Q test was used to assess the statistical heterogeneity with a *p*-value < 0.1 as a threshold for a statistically significant difference, and the I^2^ index was used to quantify between-study heterogeneity. In addition, Egger’s test and funnel plots were applied to report and visualize publication bias if at least ten studies were involved in the analysis. To assess the source of heterogeneity we performed multiple sensitivity analyses (leave-one-out meta-analysis, Baujat plot, and influence diagnostics).

A subgroup analysis was carried out with data on study design (RCT and non-RCT) and the type of screening method.

Besides heterogeneity, a *p*-value < 0.05 was considered statistically significant. Further details of the synthesis are included in the eMaterial.

### Ethical approval

No ethical approval was required for this systematic review with meta-analysis, as all data were already published in peer-reviewed journals. In addition, no patients were involved in our study’s design, conduct, or interpretation. The datasets used in this study can be found in the full-text articles included in the systematic review and meta-analysis.

## Results

### Search and selection

**Figure 1:**
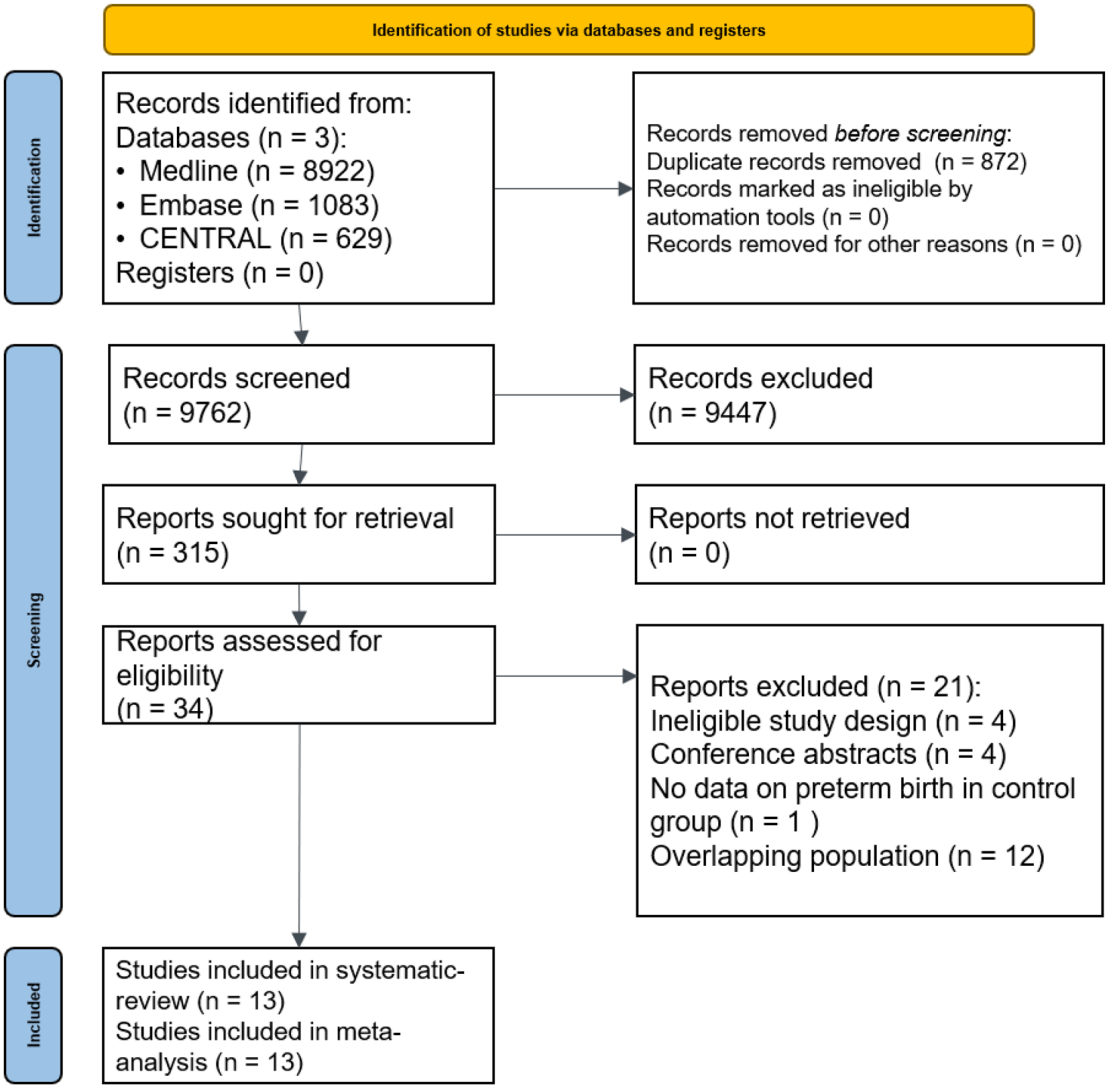
PRISMA 2020 flowchart representing the study selection process.

Altogether 10,634 studies were identified by our search key (Fig. 1). A total of 9762 studies were checked by title and abstract, and 315 by full text. After excluding overlapping populations and conference abstracts, 13 studies were eligible for systematic review and meta-analysis^10,12,19,20,27–34^.

### Baseline characteristics of included studies

Baseline characteristics of the enrolled studies are detailed in Table 1. Of the 13 included articles, nine were published from Europe, three from Asia, and one from North America. The screening methods used were different in the studies. The Nugent score evaluated the results in studies where Gram stain was used. In addition, the screening procedures’ timing and frequency among the studies were not uniform. In the intervention group, the preterm birth rate before 37 weeks ranged from 2.96 to 21.8%, while in the control group, between 5.1 and 22.3%. In most cases, oral or local Lactobacillus, Clindamycin, or Metronidazole was used to treat abnormal vaginal flora.

**Table 1:** Basic characteristics of the included studies.

Author, year	Study type	Number of patients (I/C)	Population	Preterm birth % (< 37 weeks) (I/C)	Screening period/frequency	Screening method	Treatment
Lee et al.^19^	RCT	4736/4270	Singleton and multiple pregnancies	21.8/20.6	13–19, 28–32 weeks/twice per pregnancy	Gram stain	2 × 300 mg Clindamycin per oral for 5 days
Farr et al.^12^	Retrospective cohort	8490/8651	High risk	9.7/22.3	10–16 weeks/once per pregnancy	Gram stain	Lactobacillus for 6 days in addition to Kiss et al. 2002
Bitzer et al.^12^	Prospective controlled trial	17,358/69,432	Singleton and multiple pregnancies	7.97/7.52	12–32 weeks/twice a week	pH self-testing	Diagnostic or therapeutic decisions were at the discretion of the treating gynecologist
Dennemark et al.^33^	Prospective controlled trial	600/300	Low risk	8.5/15.0	14 weeks/once per pregnancy	Gram stain	A: Clindamycin 2% vaginal cream once daily for 6 days, B: Lactobacillus once daily for 6 days
Hoyme et al.^27^	Prospective cohort	793/807	Low risk	9.2/10.9	12-delivery/50 times per pregnancy	pH self-testing	Depends on the gynecologist: Lactobacillus for 6 or 12 days, Clindamycin vaginal cream for 5 days, hospital care
Hoyme et al. (Erfurt)^10^	Prospective cohort	1382/1340	Low risk	8.9/14.6	12-delivery/50 times per pregnancy	pH test	Depends on the gynecologist: Lactobacillus for 6 or 12 days, Clindamycin vaginal cream for 5 days, hospital care
Hoyme et al. (Thuringia)^10^	Prospective cohort	8406/7870	Low risk	9.17/10.0	12 weeks-delivery/50 times per pregnancy	pH self-testing	Depends on the gynecologist: Lactobacillus for 6 or 12 days, Clindamycin vaginal cream for 5 days, hospital care
Sungkar et al.^32^	RCT	160/149	Singleton pregnancies	3.8/5.4	13 weeks-delivery/50 times per pregnancy	pH test + Gram stain	2 × 500 mg Metronidazole per os for 7 days
Batra et al.^34^	RCT	202/196	Low risk	4.5/10.7	12–28 weeks/4 weekly, 28–36 weeks/2 weekly, 36-delivery/weekly	pH test + Gram stain	2 × 400 mg Metronidazole per os for 7 days
Kiss et al.^28^	RCT	561/551	Low risk	3.9/5.1	15–19 weeks/once per pregnancy	Gram stain	Bacterial vaginosis: Clindamycin 2% vaginal cream for 6 days. Persistent or recurrent disease: 2 × 300 mg Clindamycin per oral for 7 days. Candidiasis: 100 mg local Clotrimazole for 6 days. Trichomoniasis: 500 mg local Metronidazole 7 days and included treatment of the partner
Kiss et al.^29^	RCT	2058 = 2097	Low risk	2.96/5.34	15–19 weeks/once per pregnancy	Gram stain	See Kiss et al. 2002
Kiss et al.^30^	Retrospective cohort	1273/1713	Low risk	8.17/12.6	11–24 weeks/once per gestation	Gram stain	See Kiss et al. 2002
Gjerdingen et al.^31^	RCT	63/58	Low risk	4.7/10.2	12-delivery/4 weekly (Average: 7 times)	pH test	2 × 300 mg Clindamycin per oral for 7 days, 2 × 500 mg Metronidazole per os for 7 days

*RCT* randomized controlled trial, *I/C* internvention/control.

Two studies included multiple pregnancies^19,20^. However, in these studies, the author reported the same rate of multiple pregnancies in the intervention and control groups. Furthermore, we performed a leave-one-out (sensitivity) analysis to investigate whether these studies influenced our result in a significant way, and we found no differences (eFigs. 9–14). Further details are summarized in Tables S2 and S3.

### Preterm birth before 37 weeks and birthweight under 2500 g

**Figure 2:**
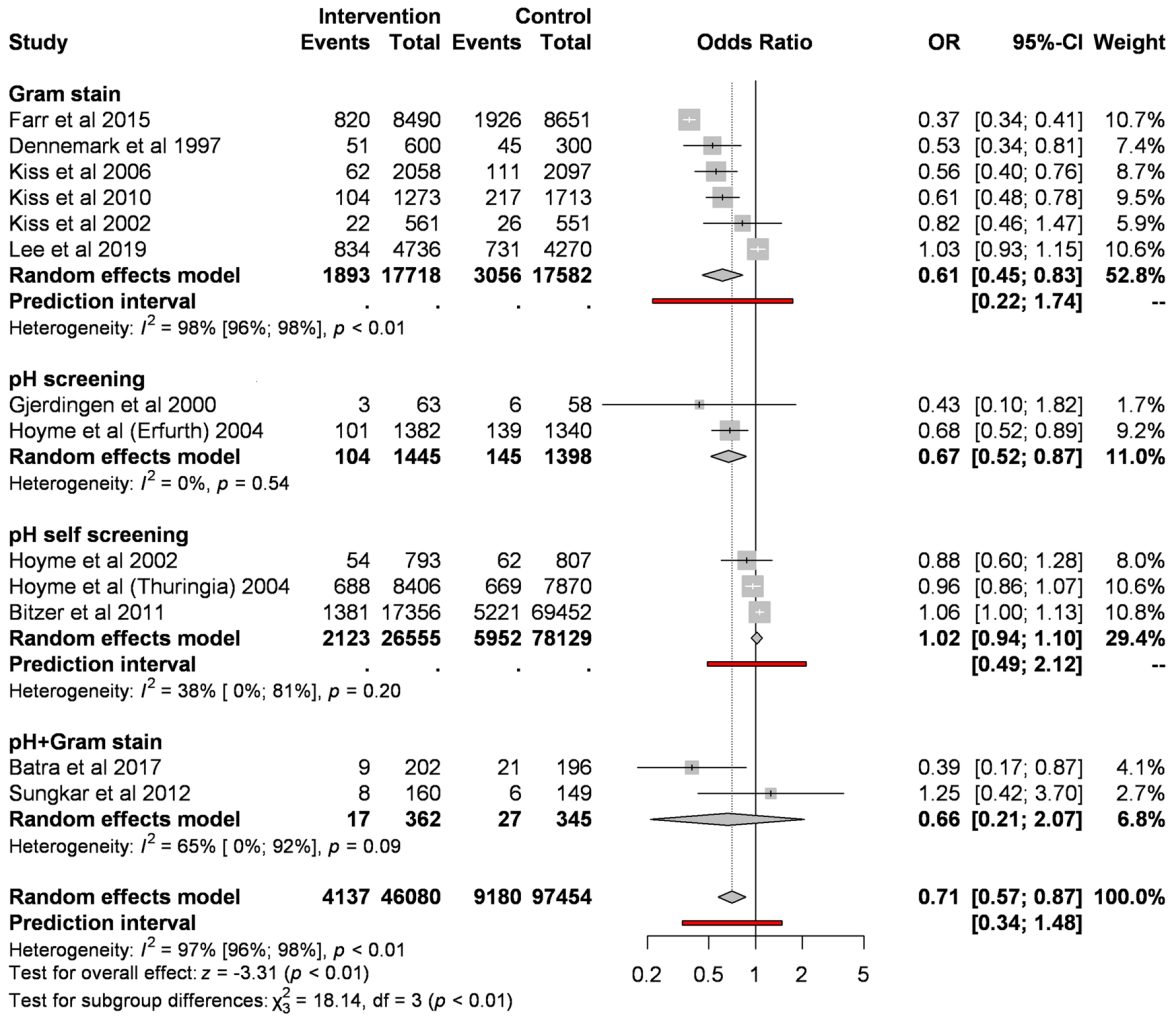
Forest plot representing the odds of preterm birth before 37 weeks.

When pooling different types of screening, regular screening of abnormal vaginal flora significantly reduces the odds of preterm birth before 37 weeks compared to control (8.98% vs. 9.42%; OR 0.71, CI 0.57–0.87, Fig. 2). However, the analysis shows substantial heterogeneity (I^2^ = 97%, p < 0.001).

Of different screening methods, Gram stain compared to control significantly reduces the odds of preterm birth before 37 weeks (10.68% vs. 17.38%; OR 0.61, CI 0.45–0.83; substantial heterogeneity). pH measurement based on two studies resulted in a similar effect (7.2% vs. 10.37%; OR 0.67, CI 0.52–0.87).

The subgroup analysis of RCTs, including all screening types, showed a tendency for lower preterm birth (OR 0.74, CI 0.53–1.03 eFig. 1), however, the difference was non-significant. The subgroup analysis of RCTs, including only Gram stain, showed similar results (OR 0.79, CI 0.54–1.16, eFig. 2).

On the other hand, screening of abnormal vaginal flora significantly reduces the odds of birthweight under 2500 g compared to control (6.53% vs. 7.24%; OR 0.64, CI 0.50–0.81, eFig. 11). However, the analysis shows substantial heterogeneity (I^2^ = 97%, p < 0.001).

Analyzing different types of screenings, Gram stain compared to control significantly reduces the odds of birthweight under 2500 g (7.94% vs. 14.79%; OR 0.55, CI 0.41–0.73; eFig. 11). Based on two studies, pH self-screening and based on one study, combined pH and Gram stain screening did not significantly differ from the control group.

The subgroup analysis of RCTs, including all screening types, showed statistically significant results for below 2500 g birthweight rate (OR 0.71, CI 0.54–0.93 eFig. 12). The subgroup analysis of RCTs, including only Gram stain, showed similar data (OR 0.71, CI 0.50–1.02 eFig. 13). However, the results were not significant.

### Preterm birth before 34 weeks and birthweight under 2000 g

Regular screening of abnormal vaginal flora reduces the odds of preterm birth before 34 weeks (3.88% vs. 4.64%; OR 0.58, CI 0.31–1.08, eFig. 7) and birthweight under 2000 g (1.94% vs. 2.81%; OR 0.49, CI 0.31–0.75, eFig. 17) compared to control. However, both analyses show substantial heterogeneity.

Based on four studies analyzing the different types of screenings, Gram stain compared to no screening significantly reduces the odds of birthweight under 2000 g (2.1% vs. 7.7%; OR 0.33, CI 0.23–0.46; substantial heterogeneity).

### Preterm birth before 32 weeks and birthweight under 1500 g

**Figure 3:**
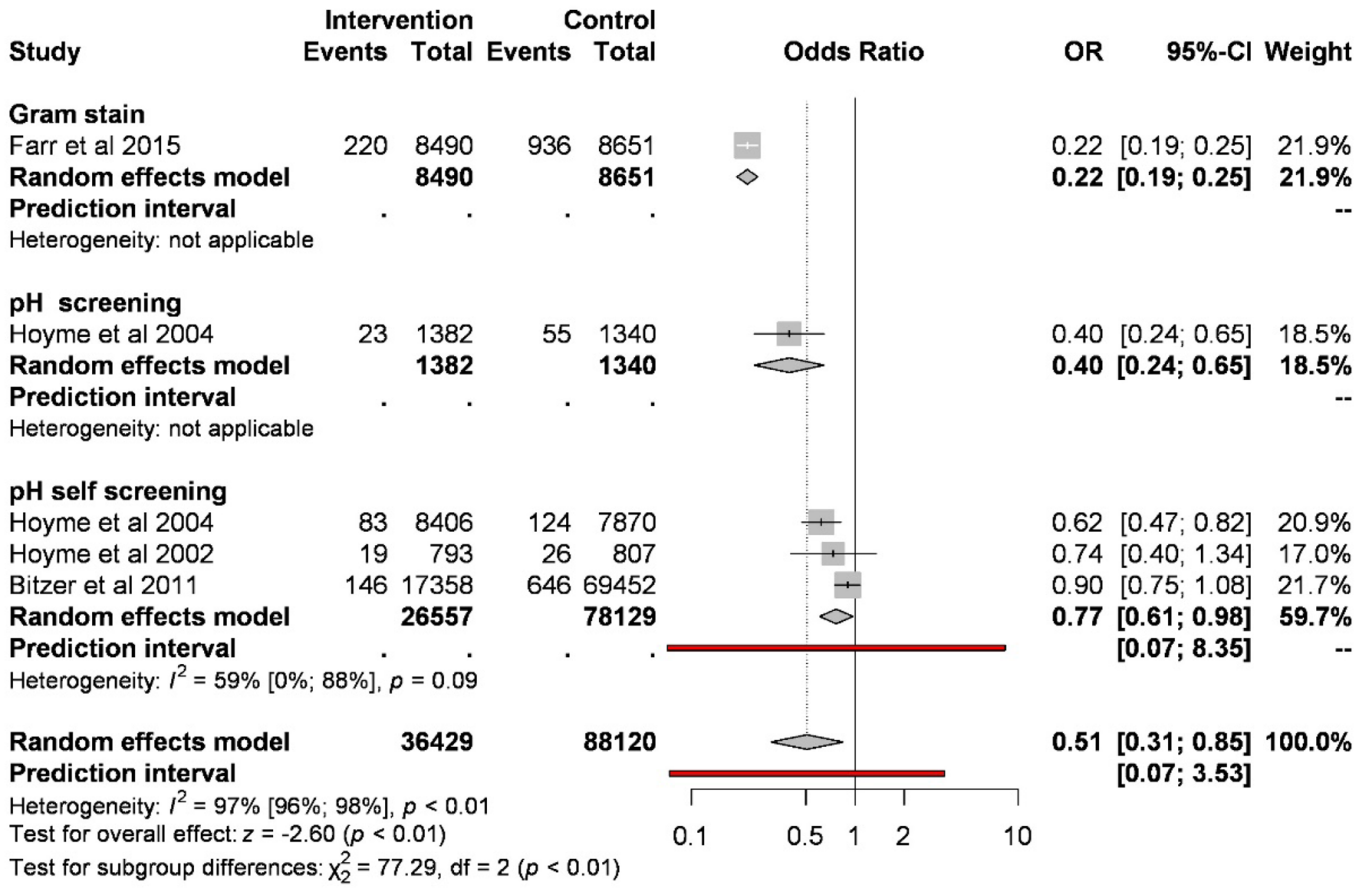
Forest plot representing the odds of preterm birth before 32 weeks.

Screening of abnormal vaginal flora significantly reduces the odds of preterm birth before 32 weeks of gestation compared to control (1.35% vs. 2.03%; OR 0.51, CI 0.31–0.85, Fig. 3). Analyzing the different types of screenings, based on three studies, pH self-screening compared to control reduces the odds of preterm birth (0.93% vs. 1.02%; OR 0.77, CI 0.61–0.98; substantial heterogeneity). Based on one study, Gram stain and pH measurement showed a similar result.

**Figure 4:**
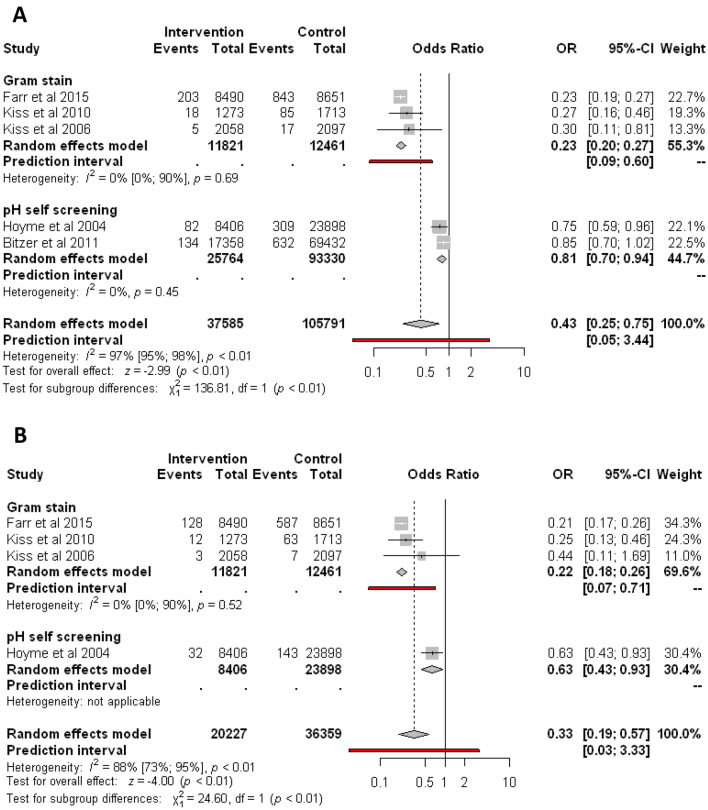
Forest plots representing the odds of bithweight under 1500 g (A) and 1000 g (B).

Similarly, regular screening of abnormal vaginal flora significantly reduces the odds of birthweight under 1500 g compared to control (1.17% vs. 1.78%; OR 0.43, CI 0.25–0.75 Fig. 4A). Analyzing the different types of screenings, Gram stain based on three studies reduces the odds of birthweight under 1500 g (1.91% vs. 7.58%; OR 0.23, CI 0.20–0.27; substantial heterogeneity). Based on two studies, pH self-screening compared to control reduces the odds of birthweight under 1500 g (0.83% vs. 1.00%; OR 0.81, CI 0.70–0.94; substantial heterogeneity).

### Birthweight under 1000 g

Regular screening of abnormal vaginal flora significantly reduces the odds of birthweight under 1000 g compared to no screening (0.86% vs. 2.2%; OR 0.33, CI 0.19–0.57, Fig. 4B). However, the analysis shows substantial heterogeneity (I^2^ = 88%, p < 0.001).

Analyzing the different types of screenings, Gram stain based on three studies significantly reduces the odds of birthweight under 1000 g (1.21% vs. 5.27%; OR 0.22, CI 0.18–0.26; substantial heterogeneity). Based on one study, pH self-screening showed a similar effect.

### Publication bias and heterogeneity

Outlier and influence analyses did not show a significant difference for any of the above mentioned results.

Egger’s test could only be performed in the case of preterm delivery before 37 weeks. For this, Eggers’ regression test shows no publication bias (t = − 0.45; df = 11; p = 0.661).

### Risk of bias assessment and level of evidence

The risk of bias assessment results is summarized in Tables S4 and S5. The overall risk of bias shows some concerns. On the other hand, the quality of evidence was very-low for most results. A summary of the assessment is included in Table S6.

## Discussion

The available evidence from our results shows that routine screening of abnormal vaginal flora during pregnancy can decrease the odds of preterm delivery. Our study is the most comprehensive on the topic, including thirteen studies with 143,534 patients worldwide.

There was a significant decrease in the odds of preterm birth before 37 and 32 weeks in the intervention group. Similarly, there was a significant decrease in the odds of delivering a newborn under 2500 g, 2000 g, and 1500 g in the intervention group. Finally, we found the most robust decrease in the odds of birthweight under 1000 g. This result has the most important clinical relevance because the chance of complications and mortality is the highest among these neonates. However, the prediction interval for most of the outcomes was wide and crossed the level of significance.

Although our study was not aimed at finding the most effective screening method, we included trials with any screening type. Based on our data, Gram stain was effective in each subgroup. Furthermore, pH self-screening, pH screening by healthcare professionals, or a combination of screening methods seemed to be effective. Interestingly, according to our data, the earlier the preterm delivery occurs, the more likely to have bacterial vaginosis in the background. In eFig. 27. we can see that as the number of weeks of gestation decreases, the greater the reduction of the odds of preterm delivery.

In different guidelines, controversial information can be found on the recommendation for routine screening of abnormal vaginal flora and treatment of bacterial vaginosis. The ACOG Practice Bulletin No. 234, published at the end of 2021, does not recommend routine screening^13^. This recommendation is based on a meta-analysis from 2013^14^. According to their results, antibiotic therapy effectively treated bacterial vaginosis. However, it did not reduce the risk of preterm birth before 37 weeks (OR 0.88; CI 0.71–1.09). On the other hand, according to the US Preventive Services Task Force Recommendation Statement, routine screening and treatment can benefit pregnant patients with a history of preterm birth^15,16^. However, the certainty of this evidence is moderate, which is why this data is insufficient for a recommendation of routine screening and treatment. It is important to mention that the ACOG recommendations against the routine screening of vaginal flora are based only on studies that investigated the efficacy or effectiveness of the treatment of bacterial vaginosis, while this review takes into account pregnant women with abnormal vaginal flora, which is why the ACOG and task force recommendations are not included in clinical trials like those of Kiss^28^.

We need to mention that the screening methods, routine pregnancy care, and treatment protocols were different in the studies as they differ from country to country. However, all screening protocols are accepted diagnostic methods. On the other hand, we performed the necessary subgroup analysis and multiple sensitivity analyses (leave-one-out meta-analysis, Baujat plot, and influence diagnostics) to exclude the potential biasing studies (eFigs. 4–6, 8–10, 14–16, 18–26.)

Another important point is the inclusion of cohort studies in our meta-analysis. Because of the low number of articles, we also decided to include both RCTs and cohort studies. Unfortunately, only a few RCTs were eligible for our study. Although we did not get significant results (OR 0.74, CI 0.53–1.03 eFig. 1) for preterm delivery (< 37 weeks), the data still suggest that routine screening might be beneficial in preventing preterm deliveries. In the case of low birthweight (< 2500 g) we got significant results in the analysis of RCTs. (OR 0.71, CI 0.54–0.93 eFig. 12).

According to the National Institute of Child Health and Human Development (NICHD), the definition of high-risk pregnancy is a pregnancy with any of the following maternal risk factors: existing health condition, malnutrition, overweight or obesity, age < 18 or > 34 years, tobacco smoking, alcohol abuse, and any condition of pregnancy such as gestational diabetes or pregnancy-induced hypertension, previous caesarean delivery, miscarriage, or preterm birth. In the study of Farr et al.^12^ a high-risk population was examined. We could see a more significant reduction in the odds of preterm birth than in low-risk population-based studies. This study seemed to be an outlier during the analysis because of the high-risk population. However, as an implication for practice, it is important to note that we can significantly reduce the odds of preterm deliveries and very or extreme preterm deliveries among high-risk pregnant women. Furthermore, we performed a leave-one-out-analysis, and we could not see a major or significant change in the results with the exclusion of this study.

It is worth mentioning that the recommendation for screening abnormal vaginal flora seems as effective as screening cervical length during the second trimester in some populations^13^. This shows that an easily executable screening method can help prevent preterm delivery^35,36^. In the case of shortened cervical length, we can choose progesterone, pessary, or cerclage as a therapy^13,37^. If we cannot postpone delivery by weeks, at least we can be prepared for preterm labor and apply prophylactic steroids to avoid the respiratory distress of premature neonates^38,39^. In the case of abnormal vaginal flora, we can use easily executable and low-cost screening and treatment methods.

Preterm birth and prematurity heavily burden the health service and the economy^40,41^. One of the included studies examined the screening for cost-effectiveness. They found that 46 euros spent on screening and treatment per person could save 56,228 euros per each prevented preterm birth^29^.

Each study used different treatment protocols (Table 1). As we talk about asymptomatic infection, some studies compared medical treatment to placebo. The general therapy for bacterial vaginosis is local or oral metronidazole or clindamycin^42,43^.

During the search process we found several important and valuable RCTs^42,44,45^. However these studies did not match our question based on the PICO framework. In these articles the efficacy of treating abnormal vaginal flora was compared to no treatment. Two of these studies^42,45^ found that treatment of abnormal vaginal flora (with oral clindamycin or metronidazole) can reduce the risk of preterm deliveries, while the third study^44^ did not prove the efficacy of oral clindamycin therapy. Therefore, there is no consensus what agent to use for the treatment of abnormal flora, or to treat it at all.

### Strengths and limitation

Regarding the strengths of our analysis, we followed a pre-defined protocol, which was registered in advance. We also applied a rigorous methodology. Another strength of our study is the low risk of bias. Additionally, the number of enrolled patients was high, and we had precisely defined outcomes. Finally, the examined screening methods are easily executable.

Considering the limitations of our study, a low number of randomized controlled studies were eligible to be enrolled. In addition, different types of screening methods were examined. Furthermore, different treatment protocols were applied for abnormal vaginal flora in the included studies, and pregnancy care protocols were different in each country. Therefore, the inclusion criteria of each study were slightly different. The different screening methods can also be considered a limitation due to their heterogeneity and different effects. However, it is important to mention that despite the different protocols, the results of the studies were very similar, which favors the hypothesis of the benefit of screening of abnormal flora. Due to the low number of studies, we could not analyze publication bias.

## Conclusion

Our systematic review found that the routine screening of abnormal vaginal flora might have a beneficial effect on the prevention of preterm birth and low birth weight deliveries. Furthermore, our results suggest that the odds of extreme preterm birth and very low birth weight delivery can also be reducedby using this method.

Moreover, our results show that any investigated screening methods, especially Gram stain, might be effective.

### Implications for practice and research

We suggest the routine screening of abnormal vaginal flora during pregnancy care based on our results. Furthermore, clinicians might consider the routine screening in the abovementioned high-risk populations.

Further randomized controlled trials are recommended for all screening methods to assess the question more accurately. The efficacy of screening methods should be compared to find the most effective one. Establishing the exact screening protocol and matching it to local pregnancy care in each country needs further investigation.

### Supplementary Information

Supplementary Information.

## Data Availability

The datasets used and/or analysed during this study are available from the corresponding author on reasonable request.
