# Supplementary material for: Routine screening of abnormal vaginal flora during pregnancy reduces the odds of preterm birth: a systematic review and meta-analysis
Source: Sci Rep. 2023 Aug 25;13:13897. doi: 10.1038/s41598-023-40993-x (PMC10457371; doi:10.1038/s41598-023-40993-x)
Supplement: Supplementary file 1 — Supplementary Information. [file 41598_2023_40993_MOESM1_ESM.docx]

**Supplementary Material**

**Title**

Routine screening of abnormal vaginal flora during pregnancy reduces the odds of preterm birth: a systematic review and meta-analysis

**Authors**

Eszter Hoffmann^1^, Alex Váradi^2^, Szilárd Váncsa^2,3,4^, Péter Hegyi^2,3,4^, Rita Nagy^2,3,4^, Balázs Hamar^1^, Vanda Futács^1^, Begüm Kepep^1^, Péter Nyirády^5^, Csaba Demendi^1^, Nándor Ács^1^

**Affiliations:**

1. Department of Obstetrics and Gynecology, Semmelweis University, Budapest, Hungary
2. Institute for Translational Medicine, Szentágothai Research Centre, Medical School, University of Pécs, Pécs, Hungary
3. Centre for Translational Medicine, Semmelweis University, Budapest, Hungary
4. Division of Pancreatic Diseases, Heart and Vascular Center, Semmelweis University, Budapest, Hungary
5. Department of Urology, Semmelweis University, Budapest, Hungary

**TABLE OF CONTENT**

**eTable 1.** PRISMA 2020 checklist

**eTable 2.** Eligibility criteria in each included article

**eTable 3.** Pregnancy care protocol and the definition of abnormal vagina flora in each included article

**eTable 4.** Risk of bias assessment using the QUIPS tools

**eTable 5.** Risk of bias assessment using the ROB2 tools

**eTable 6.** Quality of evidence using GRADEPro

**eFigure 1.** Forest plot representing the odds of preterm birth before 37 weeks by study type

**eFigure 2**. Forest plot representing the odds of preterm birth before 37 weeks by study type when the used screening method is Gram stain

**eFigure 3.** Funnel plot for Figure 2.

**eFigure 4.** Leave-one-out analysis for preterm birth before 37 weeks

**eFigure 5.** Baujat plot for preterm birth before 37 weeks

**eFigure 6.** Influence diagnostics for preterm birth before 37 weeks

**eFigure 7.** Forest plot representing the odds of preterm birth before 34 weeks

**eFigure 8.** Leave-one-out analysis for preterm birth before 32weeks

**eFigure 9.** Baujat plot for preterm birth before 32 weeks

**eFigure 10.** Influence diagnostics for preterm birth before 32 weeks

**eFigure 11.** Forest plots representing the odds of birthweight under 2500g

**eFigure 12****.** Forest plots representing the odds of birthweight under 2500g by study type

**eFigure 13. .** Forest plots representing the odds of birthweight under 2500g by study type when the used screening method is Gram stain

**eFigure 14.** Leave-one-out analysis for birthweight under 2500g

**eFigure 15.** Baujat plot for birthweight under 2500g

**eFigure 16.** Influence diagnostics for birthweight under 2500g

**eFigure 17.** Forest plots representing the odds of birthweight under 2000g

**eFigure 18.** Leave-one-out analysis for birthweight under 2000g

**eFigure 19.** Baujat plot for birthweight under 2000g

**eFigure 20.** Influence diagnostics for birthweight under 2000g

**eFigure 21.** Leave-one-out analysis for birthweight under 1500g

**eFigure 22.** Baujat plot for birthweight under 1500g

**eFigure 23.** Influence diagnostics for birthweight under 1500g

**eFigure 24.** Leave-one-out analysis for birthweight under 1000g

**eFigure 25.** Baujat plot for birthweight under 1000g

**eFigure 26.** Influence diagnostics for birthweight under 1000g

**eFigure 27.** Graphical Abstract

**eAppendix 1.** Methodology: Statistical analysis

**eTable 1.** PRISMA 2020 checklist

| **Section and topic** | **Item #** | **Checklist item** | **Location where item is reported** |  |
| --- | --- | --- | --- | --- |
| **Title** | | | | |
| Title | 1 | Identify the report as a systematic review. | 1 |  |
| **Abstract** | | | | |
| Abstract | 2 | See the PRISMA 2020 for Abstracts checklist (table 2). | 6 |  |
| **Introduction** | | | | |
| Rationale | 3 | Describe the rationale for the review in the context of existing knowledge. | 5 |  |
| Objectives | 4 | Provide an explicit statement of the objective(s) or question(s) the review addresses. | 5 |  |
| **Methods** | | | | |
| Eligibility criteria | 5 | Specify the inclusion and exclusion criteria for the review and how studies were grouped for the syntheses. | 7 |  |
| Information sources | 6 | Specify all databases, registers, websites, organisations, reference lists and other sources searched or consulted to identify studies. Specify the date when each source was last searched or consulted. | 7 |  |
| Search strategy | 7 | Present the full search strategies for all databases, registers and websites, including any filters and limits used. | 7-8 |  |
| Selection process | 8 | Specify the methods used to decide whether a study met the inclusion criteria of the review, including how many reviewers screened each record and each report retrieved, whether they worked independently, and if applicable, details of automation tools used in the process. | 8 |  |
| Data collection process | 9 | Specify the methods used to collect data from reports, including how many reviewers collected data from each report, whether they worked independently, any processes for obtaining or confirming data from study investigators, and if applicable, details of automation tools used in the process. | 8 |  |
| Data items | 10a | List and define all outcomes for which data were sought. Specify whether all results that were compatible with each outcome domain in each study were sought (e.g. for all measures, time points, analyses), and if not, the methods used to decide which results to collect. | 8 |  |
|  | 10b | List and define all other variables for which data were sought (e.g. participant and intervention characteristics, funding sources). Describe any assumptions made about any missing or unclear information. | 8 |  |
| Study risk of bias assessment | 11 | Specify the methods used to assess risk of bias in the included studies, including details of the tool(s) used, how many reviewers assessed each study and whether they worked independently, and if applicable, details of automation tools used in the process. | 8 |  |
| Effect measures | 12 | Specify for each outcome the effect measure(s) (e.g. risk ratio, mean difference) used in the synthesis or presentation of results. | 8 |  |
| Synthesis methods | 13a | Describe the processes used to decide which studies were eligible for each synthesis (e.g. tabulating the study intervention characteristics and comparing against the planned groups for each synthesis (item #5)). | 8 |  |
|  | 13b | Describe any methods required to prepare the data for presentation or synthesis, such as handling of missing summary statistics, or data conversions. | 8 |  |
|  | 13c | Describe any methods used to tabulate or visually display results of individual studies and syntheses. | 8 |  |
|  | 13d | Describe any methods used to synthesise results and provide a rationale for the choice(s). If meta-analysis was performed, describe the model(s), method(s) to identify the presence and extent of statistical heterogeneity, and software package(s) used. | 8-9 |  |
|  | 13e | Describe any methods used to explore possible causes of heterogeneity among study results (e.g. subgroup analysis, meta-regression). | 9 |  |
|  | 13f | Describe any sensitivity analyses conducted to assess robustness of the synthesised results. | 9 |  |
| Reporting bias assessment | 14 | Describe any methods used to assess risk of bias due to missing results in a synthesis (arising from reporting biases). | 8 |  |
| Certainty assessment | 15 | Describe any methods used to assess certainty (or confidence) in the body of evidence for an outcome. | 8 |  |
| **Results** | | | | |
| Study selection | 16a | Describe the results of the search and selection process, from the number of records identified in the search to the number of studies included in the review, ideally using a flow diagram (see fig 1). | 10 |  |
|  | 16b | Cite studies that might appear to meet the inclusion criteria, but which were excluded, and explain why they were excluded. | 10 |  |
| Study characteristics | 17 | Cite each included study and present its characteristics. | 10 |  |
| Risk of bias in studies | 18 | Present assessments of risk of bias for each included study. | Suppl. |  |
| Results of individual studies | 19 | For all outcomes, present, for each study: (a) summary statistics for each group (where appropriate) and (b) an effect estimate and its precision (e.g. confidence/credible interval), ideally using structured tables or plots. | Figures |  |
| Results of syntheses | 20a | For each synthesis, briefly summarise the characteristics and risk of bias among contributing studies. | 10-12 |  |
|  | 20b | Present results of all statistical syntheses conducted. If meta-analysis was done, present for each the summary estimate and its precision (e.g. confidence/credible interval) and measures of statistical heterogeneity. If comparing groups, describe the direction of the effect. | 10-12 |  |
|  | 20c | Present results of all investigations of possible causes of heterogeneity among study results. | 10-12, Suppl. |  |
|  | 20d | Present results of all sensitivity analyses conducted to assess the robustness of the synthesised results. | Suppl. |  |
| Reporting biases | 21 | Present assessments of risk of bias due to missing results (arising from reporting biases) for each synthesis assessed. | 12, Suppl. |  |
| Certainty of evidence | 22 | Present assessments of certainty (or confidence) in the body of evidence for each outcome assessed. | 12, Suppl. |  |
| **Discussion** | | | | |
| Discussion | 23a | Provide a general interpretation of the results in the context of other evidence. | 13 |  |
|  | 23b | Discuss any limitations of the evidence included in the review. | 15 |  |
|  | 23c | Discuss any limitations of the review processes used. | 15 |  |
|  | 23d | Discuss implications of the results for practice, policy, and future research. | 17 |  |
| **Other information** | | | | |
| Registration and protocol | 24a | Provide registration information for the review, including register name and registration number, or state that the review was not registered. | 7 |  |
|  | 24b | Indicate where the review protocol can be accessed, or state that a protocol was not prepared. | 7 |  |
|  | 24c | Describe and explain any amendments to information provided at registration or in the protocol. | 7 |  |
| Support | 25 | Describe sources of financial or non-financial support for the review, and the role of the funders or sponsors in the review. | 2 |  |
| Competing interests | 26 | Declare any competing interests of review authors. | 2 |  |
| Availability of data, code, and other materials | 27 | Report which of the following are publicly available and where they can be found: template data collection forms; data extracted from included studies; data used for all analyses; analytic code; any other materials used in the review. | 2 |  |

**eTable 2.** Eligibility criteria in each included article

| **Author (year)** | **Inclusion criteria** | **Exclusion criteria** |
| --- | --- | --- |
| Lee et al, 2019 ^1^ | “Eligible participants within clusters were all ever-married women and girls of reproductive age (aged 15–49 years) who became pregnant during the study period. All women and girls in the study area whose pregnancy was detected before 19 weeks’ gestation were eligible to enroll in the study.” | “Women were excluded from the study if they were unsure of the date of their last menstrual period (because of lactational amenorrhea, recent discontinuation of contraception, or irregular menses) or had severe chronic disease.” |
| Farr et al, 2015 ^2^ | “The study included retrospectively collected data from all of the women who presented with singleton pregnancies between January 1, 2005, and January 1, 2014, at our tertiary referral center. According to the National Institute of Child Health and Human Development (NICHD), a high-risk pregnancy was defined as a pregnancy with any of the following maternal characteristics: existing health condition, malnutrition, overweight or obesity, age < 18 or > 34 years, tobacco smoking, alcohol abuse, and any condition of pregnancy such as gestational diabetes or pregnancy induced hypertension, previous cesarean delivery, miscarriage, or preterm birth. The study intervention group consisted of all of the women who registered for a planned birth at our department between 10 + 0 (10 weeks plus 0 days) and 16 + 0 (16 weeks plus 0 days) weeks of gestation and consequently underwent the antenatal infection screen-and-treat program. The control group included all of the women with singleton pregnancies who delivered at our department over the same time period and who did not undergo the antenatal infection screen-and-treat program because of refusal of the intervention, birth registration before 10 + 0 or after 16 + 0 gestational weeks.” | “As part of our routine protocol, women at low or moderate risk were referred to other hospitals.” |
| Bitzer et al, 2011 ^3^ | “From 2004 to 2006, in a model project carried out by four German health insurers, expectant mothers were offered self-testing of vaginal pH in order to prevent preterm delivery. Only births for which definite matching of mother with child was possible, and for which information about both mother and child was available from both data sources, were analyzed.” | No information |
| Dennemark et al, 1997 ^4^ | “Nine hundred pregnant women were enrolled in three neighbouring gynaecological practices (A, B and C) in Berlin. Each practice continuously recruited 300 women with singleton pregnancies at their first antenatal visit.” | No information |
| Hoyme et al, 2002 ^5^ | “The patients were recruited into the program with the help of a simple explanatory leaflet at the end of the 12th week of pregnancy. Women who were at least 12+0 weeks pregnant and whose delivery date was 14 September 2000 or later were recruited for the campaign.” | No information |
| Hoyme et al, 2004 (Erfurt) ^6^ | “The patients were recruited into the program with the help of a simple explanatory leaflet at the end of the 12th week of pregnancy.” | No information |
| Hoyme et al, 2004 (Thuringia) ^6^ | “The objective of the trial was to involve all pregnant women throughout the federal state (about 16,000 per year) for a defined period of time. Women who were at least 12+0 weeks pregnant and whose delivery date was 14 September 2000 or later were recruited for the campaign.” | No information |
| Sungkar et al, 2012 ^7^ | “Eligible participants were pregnant women (nulliparous and multiparous) with singleton pregnancies (14–18 weeks), making their first visit prenatal visit to 1 of 5 prenatal clinics across Jakarta or Cipto Mangunkusumo National Central Hospital, Jakarta, Indonesia.” | Pregnant women with known multiple pregnancies, known fetal anomalies, or uterine malformation were ineligible to participate in the study. |
| Batra et al, 2017 ^8^ | “All the pregnant women with gestational age between 14 to 20 weeks attending the antenatal clinic, who were willing to participate in the study, were included till the required sample size of 440 was obtained.” | “Women with multiple gestation, oligohydramnios, medical disorders (cardiac, respiratory, renal, liver, and endocrine), asymptomatic bacteriuria, and vaginal bleeding were excluded from study.” |
| Kiss et al, 2002 ^9^ | “Pregnant women presenting for their routine prenatal visits between 15+0 (15 weeks plus 0 days) and 19+6 weeks (19 weeks plus 6 days) of gestation. The obstetricians included women without subjective complaints (contractions, vaginal bleeding, or symptoms suggestive of vaginal infection) into the study after obtaining individual informed consent.” | “We excluded women erroneously included—those who did not fulfil the inclusion criteria in terms of week of gestation or clinical symptoms of vaginal infection, or those with multiple pregnancies.” |
| Kiss et al, 2006 ^10^ | “Pregnant women presenting for their routine prenatal visits between 15+0 (15 weeks plus 0 days) and 19+6 weeks (19 weeks plus 6 days) of gestation. The obstetricians included women without subjective complaints (contractions, vaginal bleeding, or symptoms suggestive of vaginal infection) into the study after obtaining individual informed consent.” | “We excluded women erroneously included—those who did not fulfil the inclusion criteria in terms of week of gestation or clinical symptoms of vaginal infection, or those with multiple pregnancies.” |
| Kiss et al, 2010 ^11^ | “Data of all women presenting with singleton pregnancies between 11 + 0 weeks (11 weeks plus 0 days) and 24 + 6 weeks of gestation and registering for delivery at the Department of Obstetrics and Gynaecology of the University of Vienna between 1 September 2004 and 31 August 2005 were retrospectively collected for the intervention group of this study. The historical control group consisted of all women with singleton pregnancies between 11 + 0 weeks and 24 + 6 weeks of gestation having registered for delivery at our department 2 years previously, i.e., between 1 September 2002 and 31 August 2003.” | No information |
| Gjerdingen et al, 2000 ^12^ | “We enrolled pregnant women of less than 34 weeks of gestation attending 1 of 3 participating clinics located in the Minneapolis/St. Paul, Minnesota, metropolitan area between July 1, 1996, and December 31, 1997.” | No information |

**eTable 3.** Pregnancy care protocol and the definition of abnormal vagina flora in each included article

| **Author (year)** | **Pregnancy care protocol** | **Definition of abnormal vaginal flora** | **Gestational age determination** |
| --- | --- | --- | --- |
| Lee et al, 2019 ^1^ | “Community health workers provided basic home-based antenatal care (between 13 and 19 weeks’ and 28 and 32 weeks’ gestation)37 in all study areas.” | “Vaginal swab samples were Gram stained and Nugent scored.” | “Gestational age was based on the first day of the last menstrual period.” |
| Farr et al, 2015 ^2^ | “Prenatal care was equal in both study groups as routine consultations were performed in obstetric offices with documentation in the official booklet according to the Austrian government’s welfare program.” | “Our program protocol follows the classification of the vaginal bacterial flora by Nugent et al, 1991. ^13^” | No information |
| Bitzer et al, 2011 ^3^ | No information | “Mothers were offered self-testing of vaginal pH. They were instructed to consult with a gynecologist after any positive result.” | No information |
| Dennemark et al, 1997 ^4^ | “All routine antenatal examinations, including a syphilis test and screening for Chlamydia trachomatis, were completed according to national pregnancy care guidelines.” | “Bacterial vaginosis was determined by the presence of `clue cells’ on the wet mount preparation. Clinical criteria such as fishy odour and thin, grey, homogeneous discharge were recorded.” | “Gestational age was determined by ultrasound examination.” |
| Hoyme et al, 2002 ^5^ | No information | “The sensitivity of a pH >4:7 with respect to bacterial vaginosis is 97%; a pH value >4.7 is only 67% specific and not sensitive for other infections.” | No information |
| Hoyme et al, 2004 (Erfurt) ^6^ | No information | “The sensitivity of a pH >4:7 with respect to bacterial vaginosis is 97%; a pH value >4.7 is only 67% specific and not sensitive for other infections.” | No information |
| Hoyme et al, 2004 (Thuringia) ^6^ | No information | “The sensitivity of a pH >4:7 with respect to bacterial vaginosis is 97%; a pH value >4.7 is only 67% specific and not sensitive for other infections.” | No information |
| Sungkar et al, 2012 ^7^ | Every 4 week (12-30 weeks), Fornightly (30-36 weeks), Weekly (36 weeks-delivery) | “pH paper: A positive result was assumed when the color change recorded after 15 seconds was similar to the positive reference color on the reference card provided. Vaginal swab for Gram stain was taken from the participants who had a positive result on the self-examination kit and those with symptomatic complaints. Bacterial vaginosis was diagnosed by Amsel criteria.” | No information |
| Batra et al, 2017 ^8^ | No information | “pH of >4.5 was classified as presence of bacterial vaginosis. Another swab sample was screened by Nugent score.” | No information |
| Kiss et al, 2002 ^9^ | No information | “To diagnose bacterial vaginosis we Gram stained all preparations in a central laboratory and used the scoring system proposed by Nugent et al for our evaluation. The study protocol differentiated between bacterial vaginosis (Nugent grade 3), vaginal candidiasis (spores and hyphae), infection with *T vaginalis*, or combinations of any of the three.” | “Patients' obstetricians determined gestational age on the basis of the date of a woman's last menstrual period and confirmed this by ultrasound before 19 weeks of gestation.” |
| Kiss et al, 2006 ^10^ | No information | “To diagnose bacterial vaginosis we Gram stained all preparations in a central laboratory and used the scoring system proposed by Nugent et al for our evaluation. The study protocol differentiated between bacterial vaginosis (Nugent grade 3), vaginal candidiasis (spores and hyphae), infection with *T vaginalis*, or combinations of any of the three.” | “Patients' obstetricians determined gestational age on the basis of the date of a woman's last menstrual period and confirmed this by ultrasound before 19 weeks of gestation.” |
| Kiss et al, 2010 ^11^ | No information | “For the diagnosis of bacterial vaginosis, all preparations were Gram-stained and evaluated by the scoring system proposed by Nugent et al. [19], which classifies into normal (grade 1), intermediate (grade 2), and bacterial vaginosis (grade 3). The study protocol differentiated between bacterial vaginosis (Nugent grade 3), vaginal candidiasis (spores and hyphae), infection with T. vaginalis, or combinations of any of the three.” | “The gestational age was determined based on the date of the last menstrual period and ultrasonography.” |
| Gjerdingen et al, 2000 ^12^ | No information | “Women with a pH level >4.5 also had a wet mount examination performed.” | No information |

**eTable 4.** Summary of the risk of bias assessment (ROBINS I)


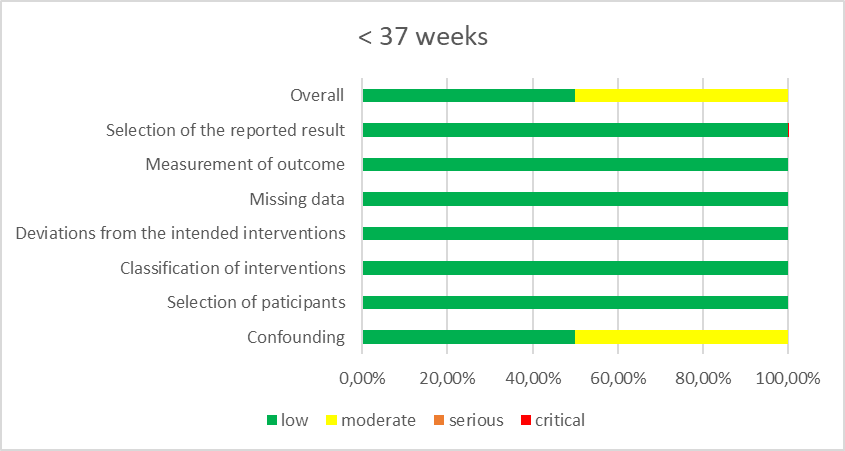


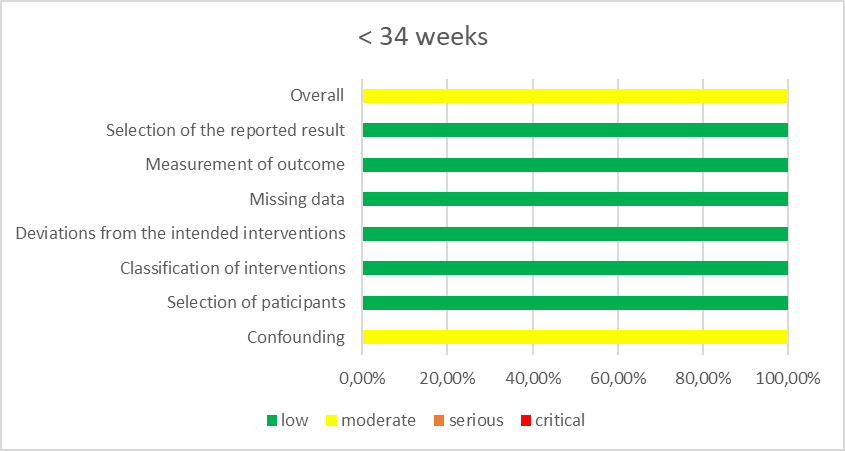


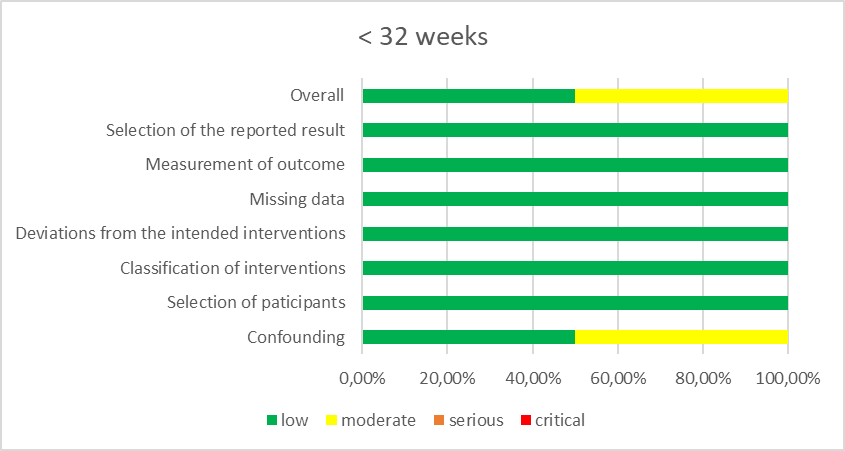


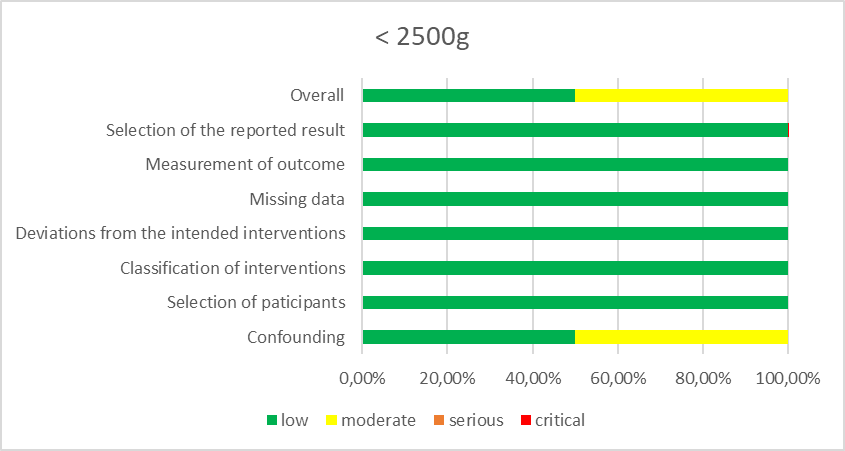


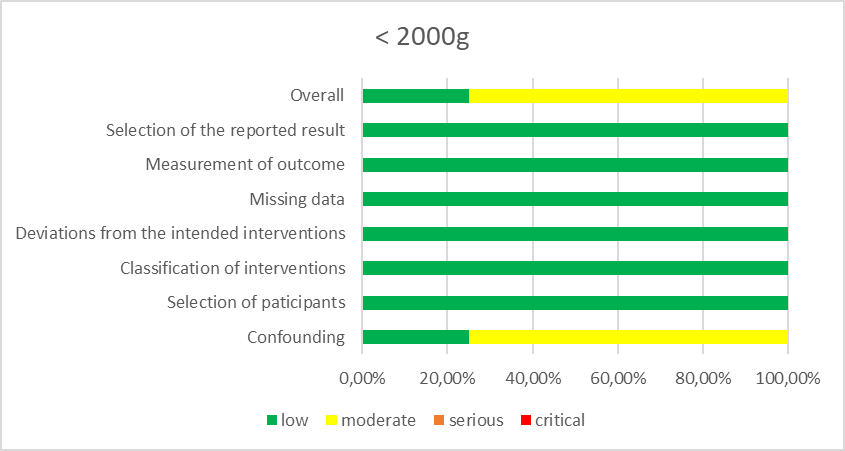


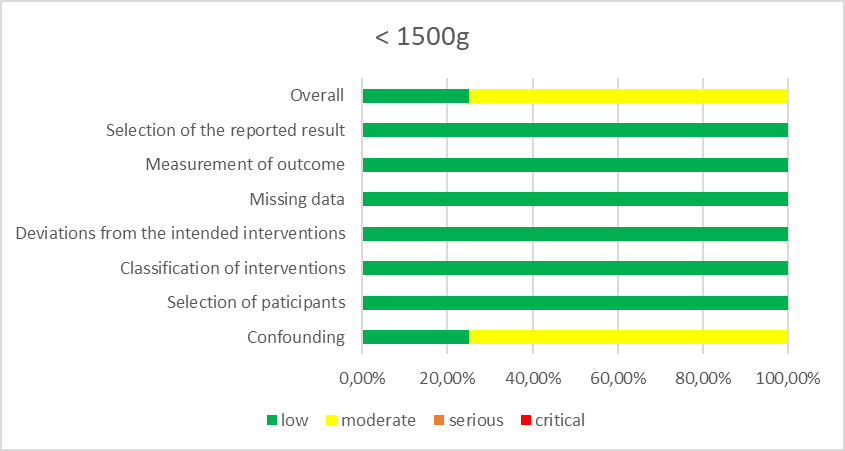


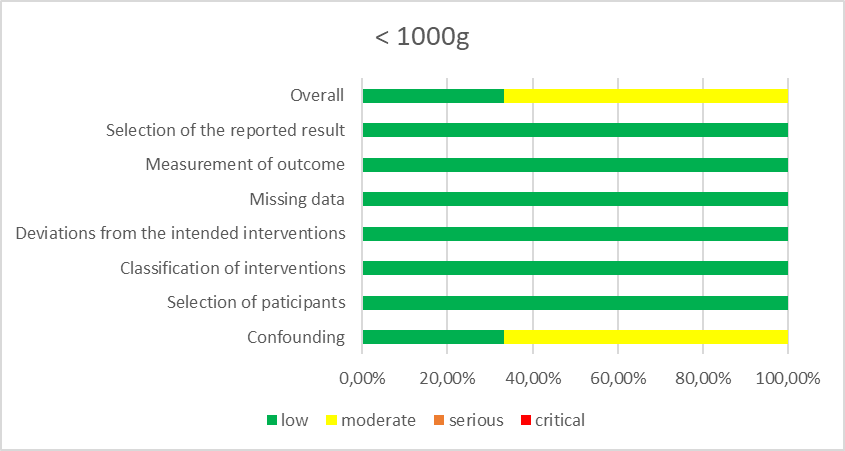


**eTable 5.** Risk of bias assessment using the ROB2 tools


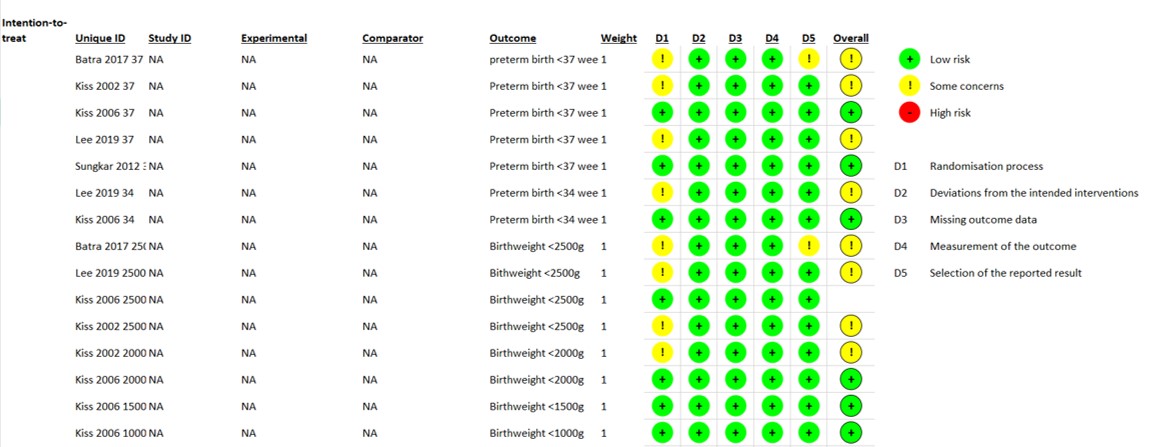


**eTable 6.** Quality of evidence using GRADEPro

| **Screening compared to no screening for prevention of preterm birth** | | | | | |
| --- | --- | --- | --- | --- | --- |
| **Outcomes** | **№ of participants (studies) Follow-up** | **Certainty of the evidence (GRADE)** | **Relative effect (95% CI)** | **Anticipated absolute effects** | |
|  |  |  |  | **Risk with no screening** | **Risk difference with screening** |
| < 37 weeks RCT | 15101 (6 RCTs) | ⨁⨁◯◯ Low | **OR 0.74** (0.53 to 1.03) | 123 per 1 000 | **29 fewer per 1 000** (54 fewer to 3 more) |
| < 37 weeks Observational studies | 128433 (7 observational studies) | ⨁◯◯◯ Very low | **OR 0.90** (0.86 to 0.94) | 92 per 1 000 | **8 fewer per 1 000** (12 fewer to 5 fewer) |
| < 37 weeks overall | 142533 (13 observational studies) | ⨁◯◯◯ Very low | **OR 0.71** (0.58 to 0.84) | 94 per 1 000 | **25 fewer per 1 000** (37 fewer to 14 fewer) |
| < 34 weeks | 16147 (3 RCTs) | ⨁⨁◯◯ Low | **OR 0.58** (0.31 to 1.08) | 46 per 1 000 | **19 fewer per 1 000** (32 fewer to 4 more) |
| < 32 weeks | 124549 (5 observational studies) | ⨁◯◯◯ Very low | **OR 0.51** (0.31 to 0.85) | 20 per 1 000 | **10 fewer per 1 000** (14 fewer to 3 fewer) |
| < 2500g RCT | 14071 (4 RCTs) | ⨁⨁◯◯ Low | **OR 0.71** (0.54 to 0.93) | 96 per 1 000 | **26 fewer per 1 000** (42 fewer to 6 fewer) |
| < 2500g Observational studies | 140121 (5 observational studies) | ⨁◯◯◯ Very low | **OR 0.94** (0.89 to 0.99) | 71 per 1 000 | **4 fewer per 1 000** (7 fewer to 1 fewer) |
| < 2500g overall | 154192 (13 observational studies) | ⨁◯◯◯ Very low | **OR 0.64** (0.50 to 0.81) | 72 per 1 000 | **25 fewer per 1 000** (35 fewer to 13 fewer) |
| < 2000g | 144488 (6 observational studies) | ⨁◯◯◯ Very low | **OR 0.49** (0.31 to 0.75) | 28 per 1 000 | **14 fewer per 1 000** (19 fewer to 7 fewer) |
| < 1500g | 143376 (5 observational studies) | ⨁◯◯◯ Very low | **OR 0.43** (0.25 to 0.75) | 18 per 1 000 | **10 fewer per 1 000** (13 fewer to 4 fewer) |
| < 1000g | 56586 (4 observational studies) | ⨁◯◯◯ Very low | **OR 0.33** (0.19 to 0.57) | 22 per 1 000 | **15 fewer per 1 000** (18 fewer to 9 fewer) |
| Gram stain, < 37 weeks RCT | 14273 (3 RCTs) | ⨁⨁◯◯ Low | **OR 0.79** (0.54 to 1.16) | 125 per 1 000 | **24 fewer per 1 000** (54 fewer to 17 more) |
| Gram stain, < 37 weeks Observational studies | 21027 (3 observational studies) | ⨁◯◯◯ Very low | **OR 0.48** (0.35 to 0.65) | 205 per 1 000 | **95 fewer per 1 000** (122 fewer to 61 fewer) |
| Gram stain, < 37 weeks overall | 35301 (6 observational studies) | ⨁◯◯◯ Very low | **OR 0.61** (0.45 to 0.83) | 174 per 1 000 | **60 fewer per 1 000** (87 fewer to 25 fewer) |
| Gram stain, < 2500g RCT | 13673 (3 RCTs) | ⨁⨁◯◯ Low | **OR 0.71** (0.50 to 1.02) | 95 per 1 000 | **26 fewer per 1 000** (45 fewer to 2 more) |
| Gram stain, < 2500g Observational studies | 21027 (3 observational studies) | ⨁◯◯◯ Very low | **OR 0.41** (0.33 to 0.51) | 180 per 1 000 | **97 fewer per 1 000** (112 fewer to 79 fewer) |
| Gram stain, < 2500g overall | 34700 (6 observational studies) | ⨁◯◯◯ Very low | **OR 0.55** (0.41 to 0.73) | 142 per 1 000 | **59 fewer per 1 000** (79 fewer to 34 fewer) |


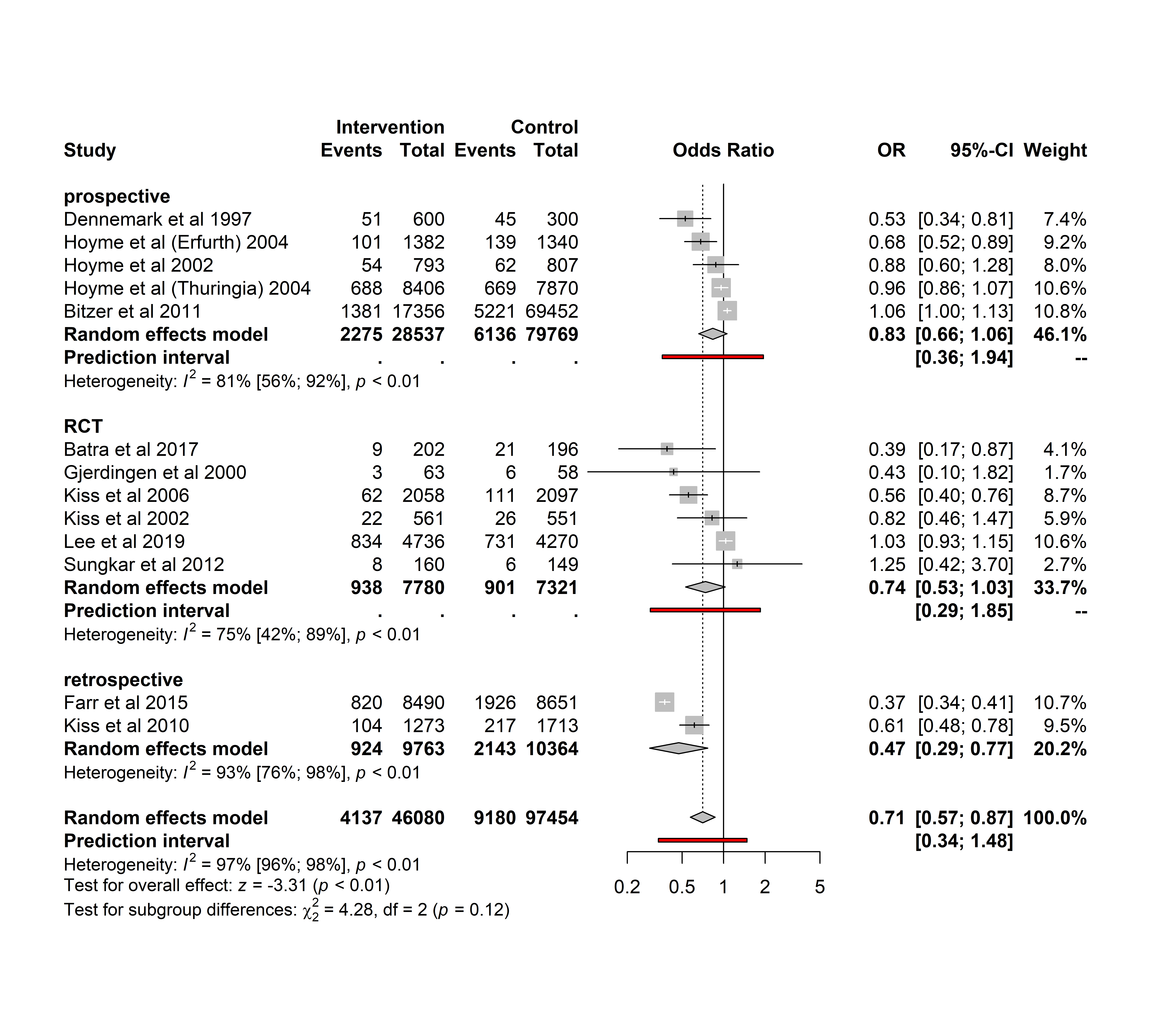
**eFigure 1.** Forest plot representing the odds of preterm birth before 37 weeks by study type

**eFigure 2**. Forest plot representing the odds of preterm birth before 37 weeks
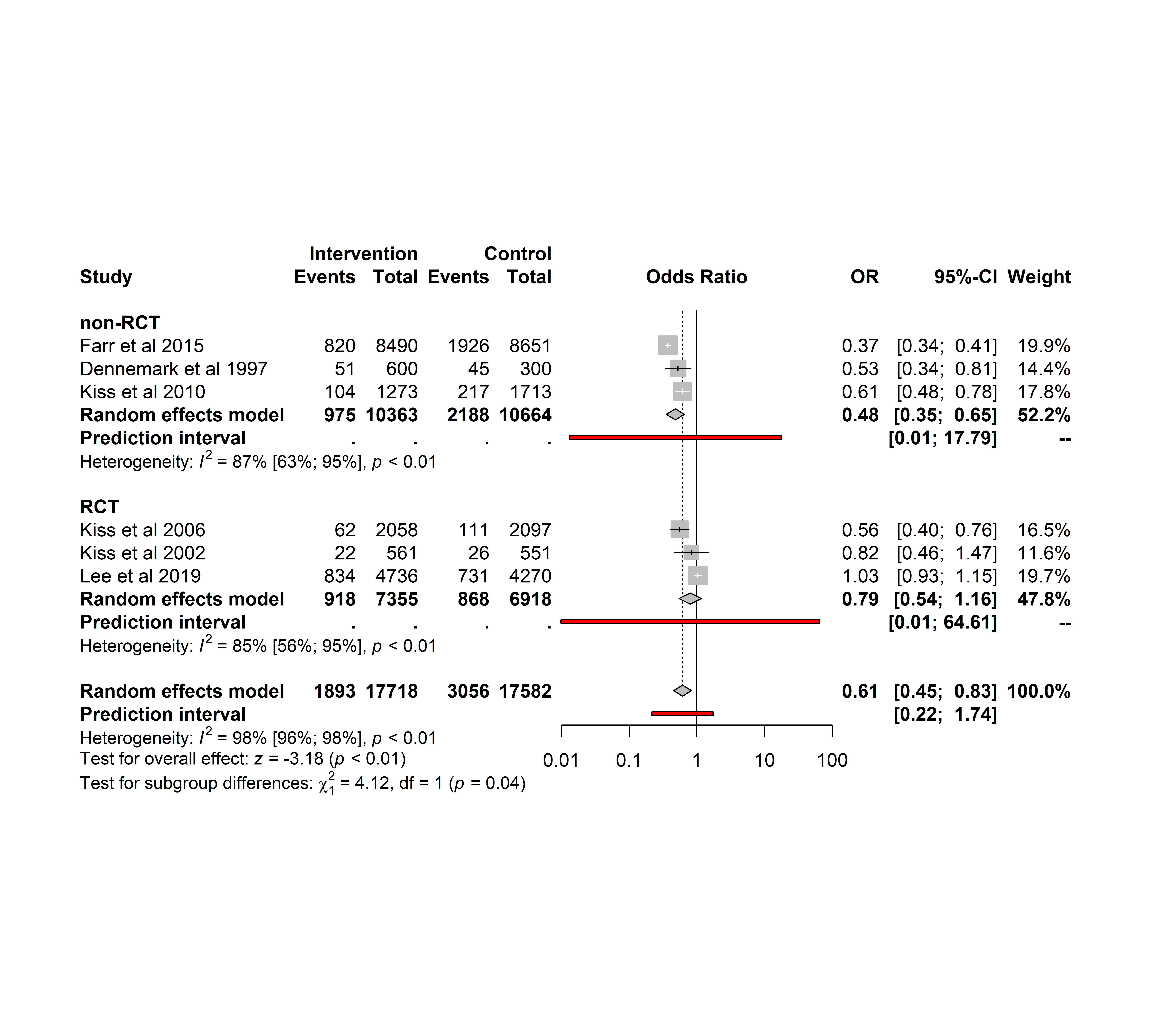
 by study type when the used screening method is Gram stain

**eFigure 3.** Funnel plot for Figure 2.


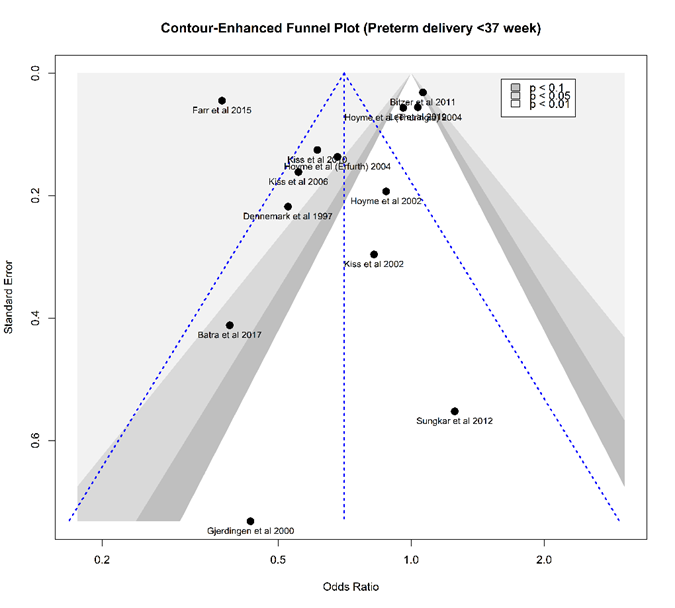


**eFigure 4.** Leave-one-out analysis for preterm birth before 37 weeks


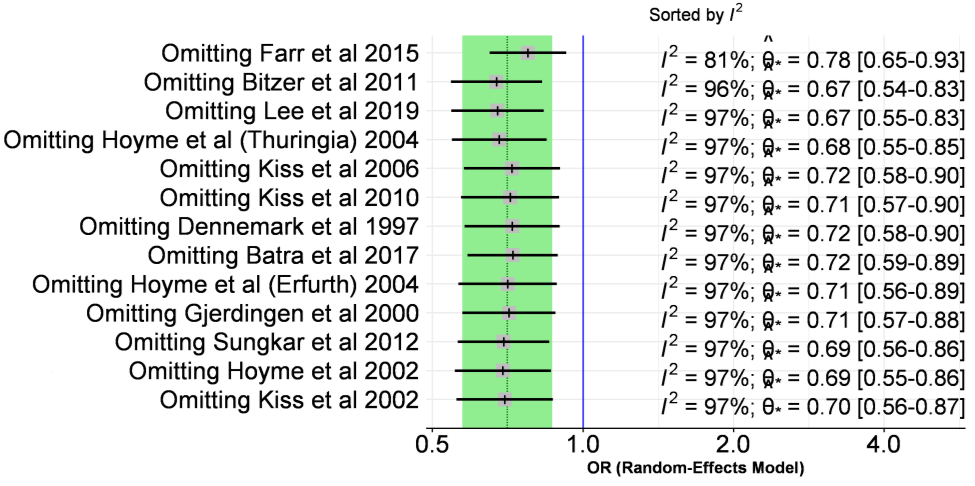


**eFigure 5.** Baujat plot for preterm birth before 37 weeks


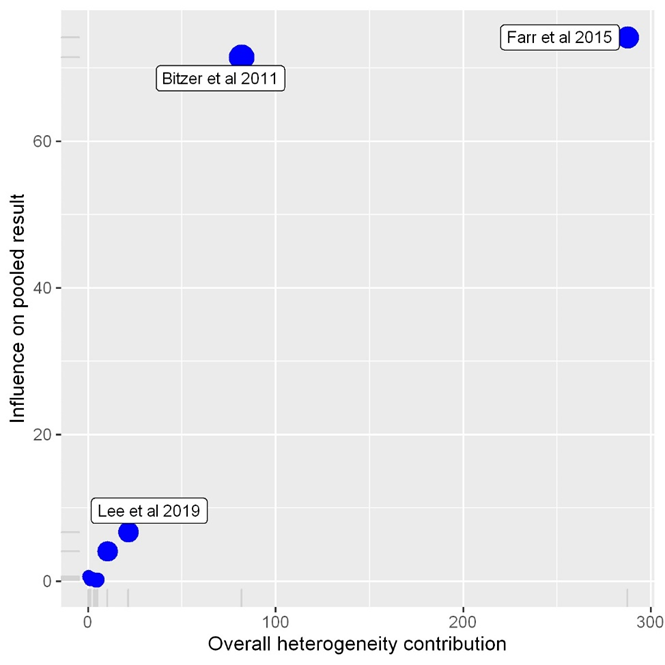


**eFigure 6.** Influence diagnostics for preterm birth before 37 weeks

**
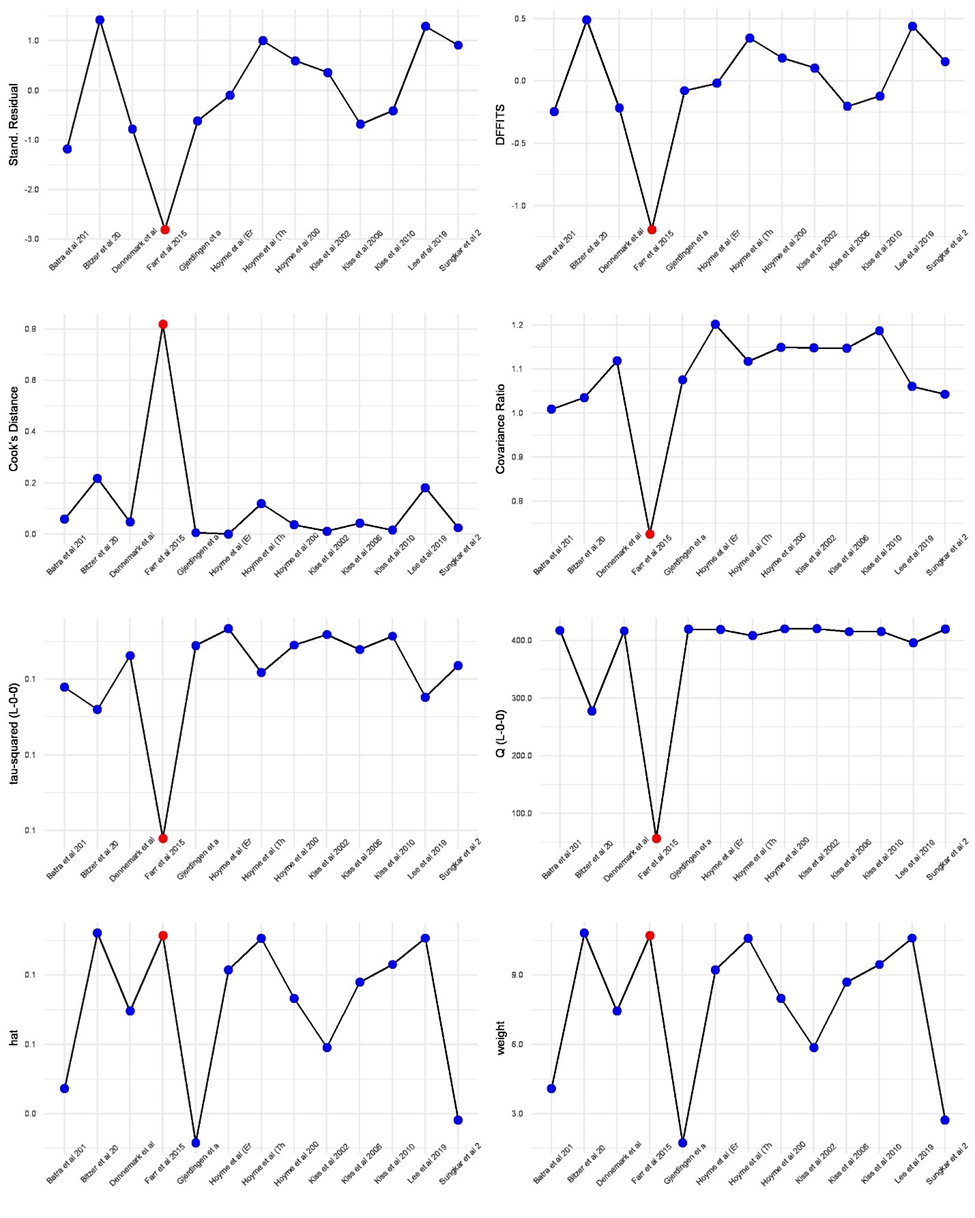
**

**eFigure 7.** Forest plot representing the odds of preterm birth before 34 weeks


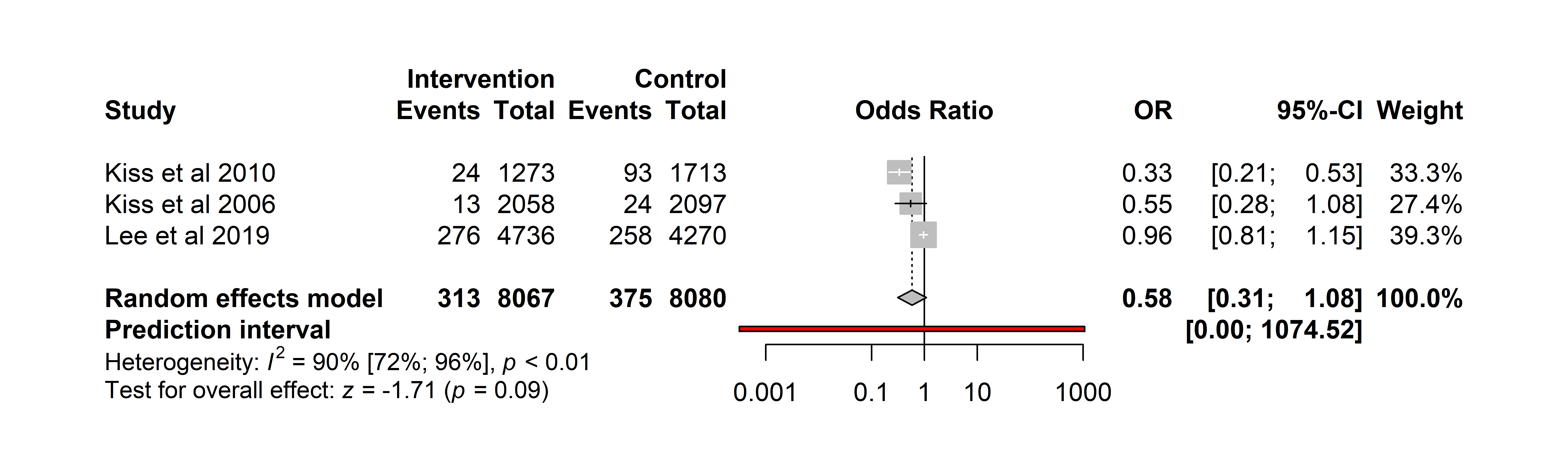


**eFigure 8.** Leave-one-out analysis for preterm birth before 32 weeks


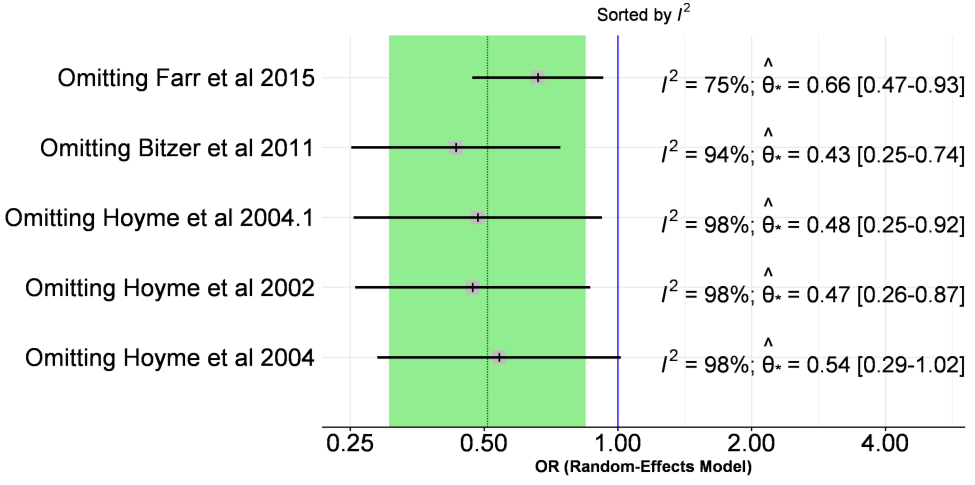


**eFigure 9.** Baujat plot for preterm birth before 32 weeks

**
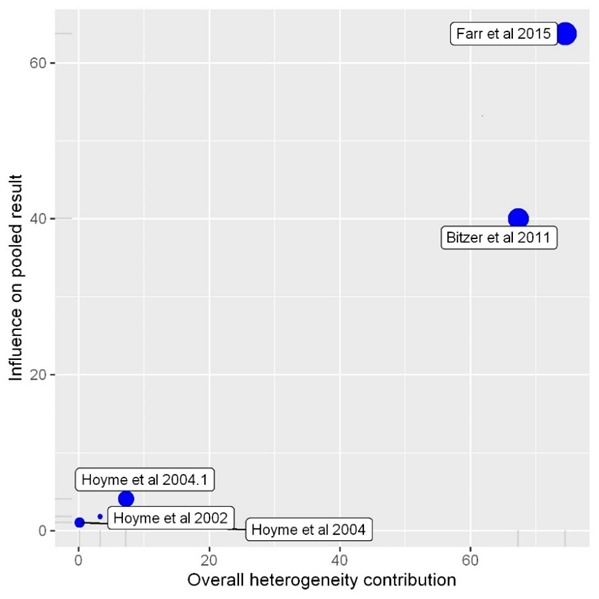
**

**eFigure 10.** Influence diagnostics for preterm birth before 32 weeks

**
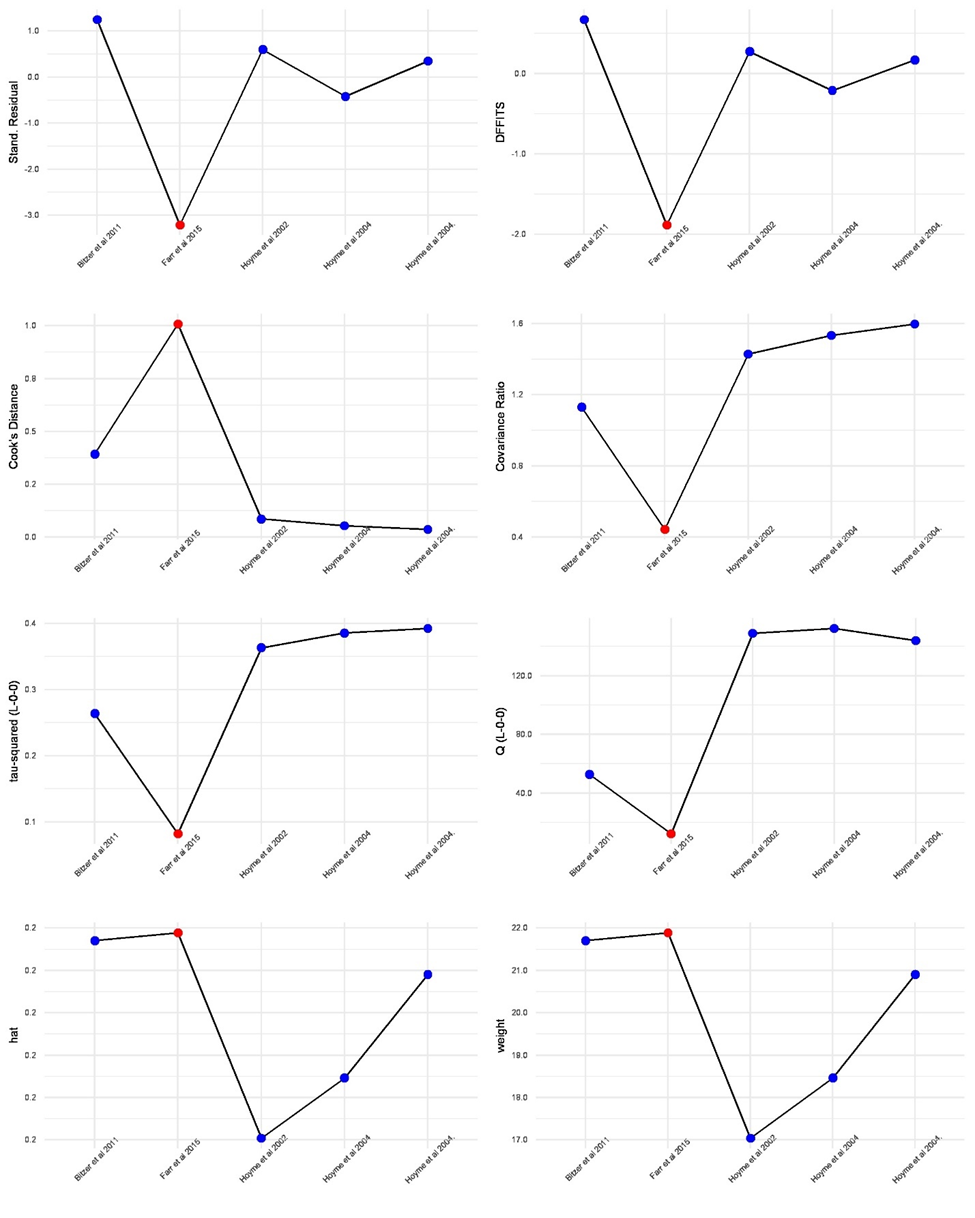
**

**eFigure 11.** Forest plot representing the odds of birthweight under 2500g


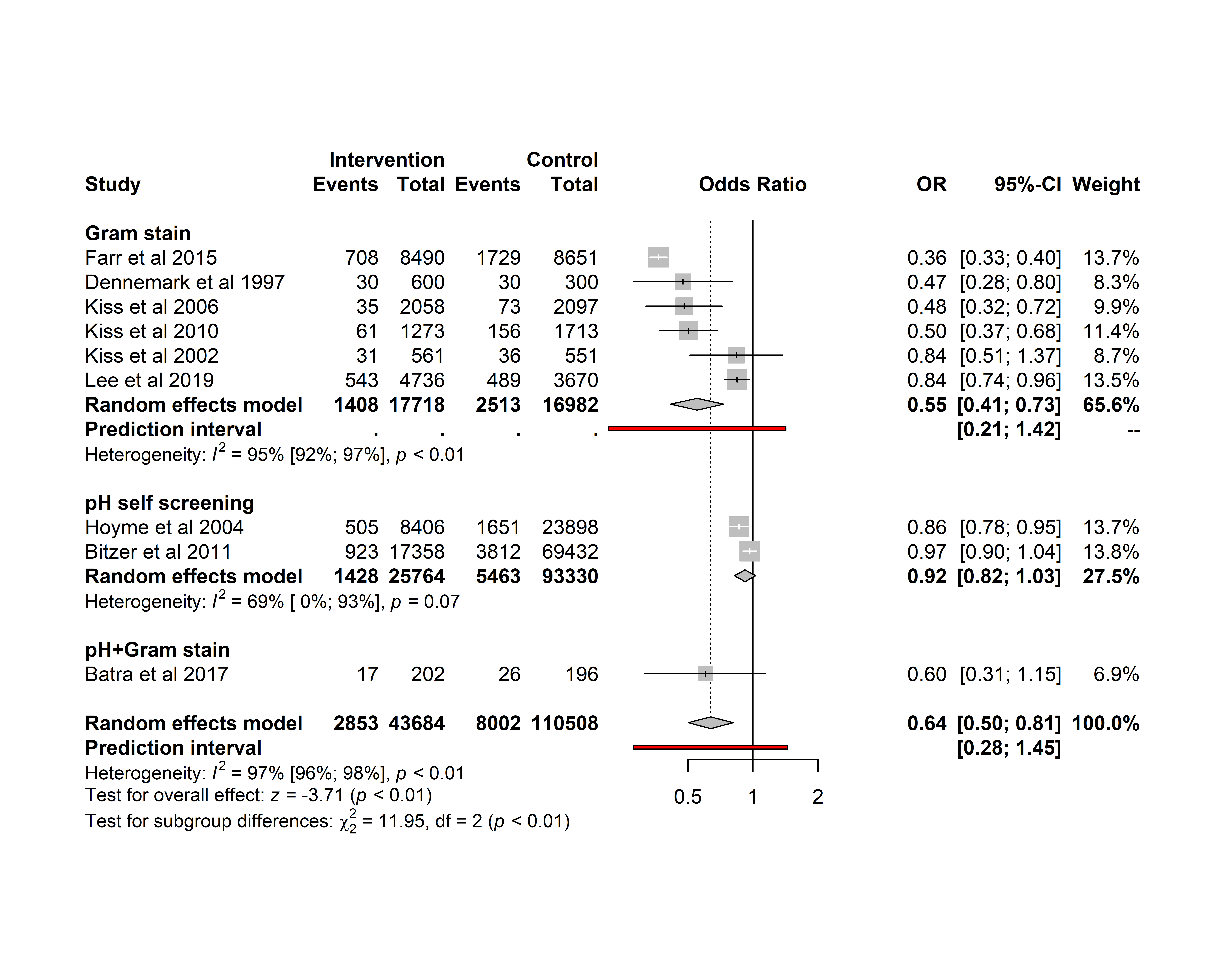


**eFigure 12.** Forest plot representing the odds of birthweight under 2500g by study type


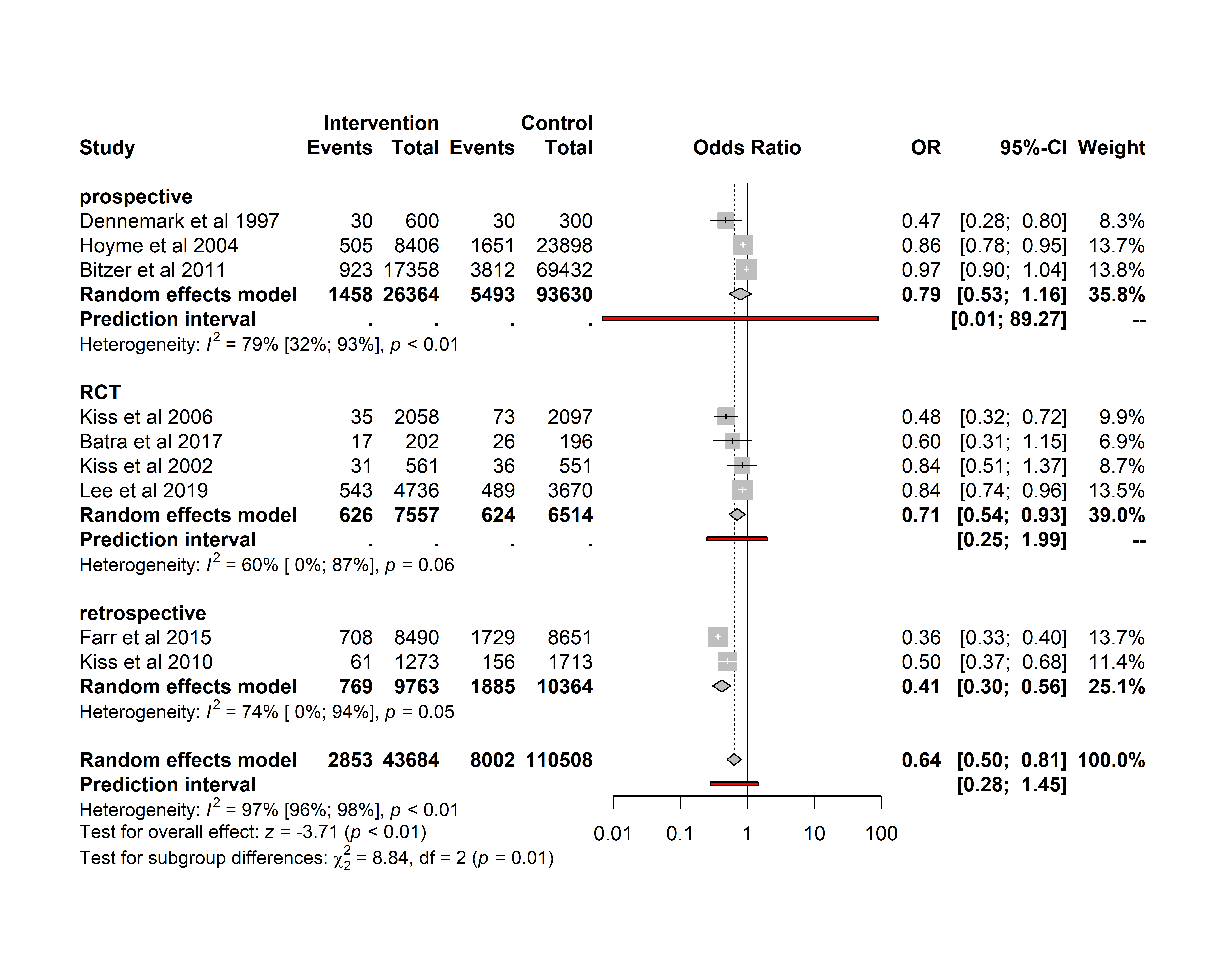


**eFigure 13.** Forest plot representing the odds of birthweight under 2500g by study type when the used screening method is Gram stain


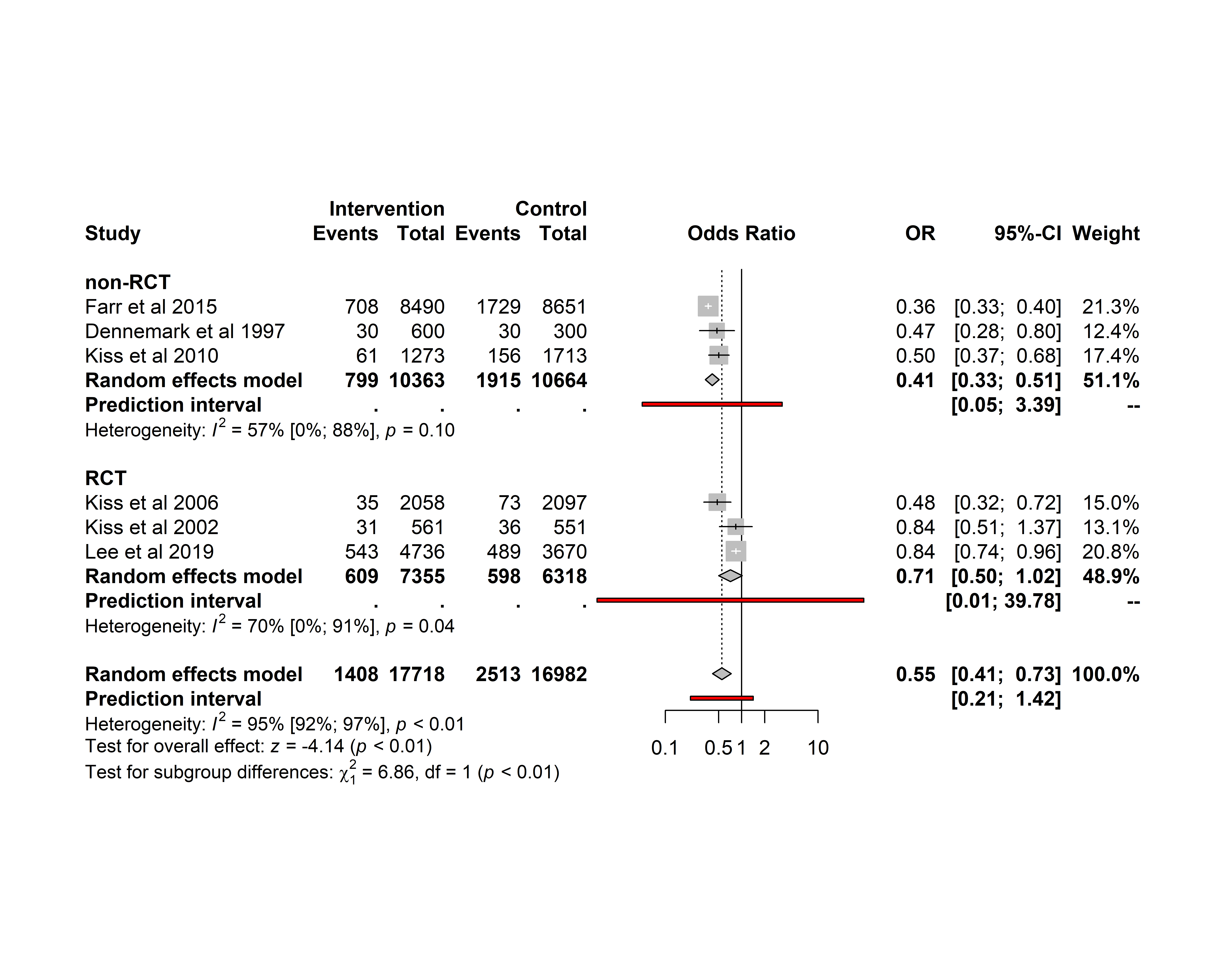


**eFigure 14.** Leave-one-out analysis for birthweight under 2500g

**
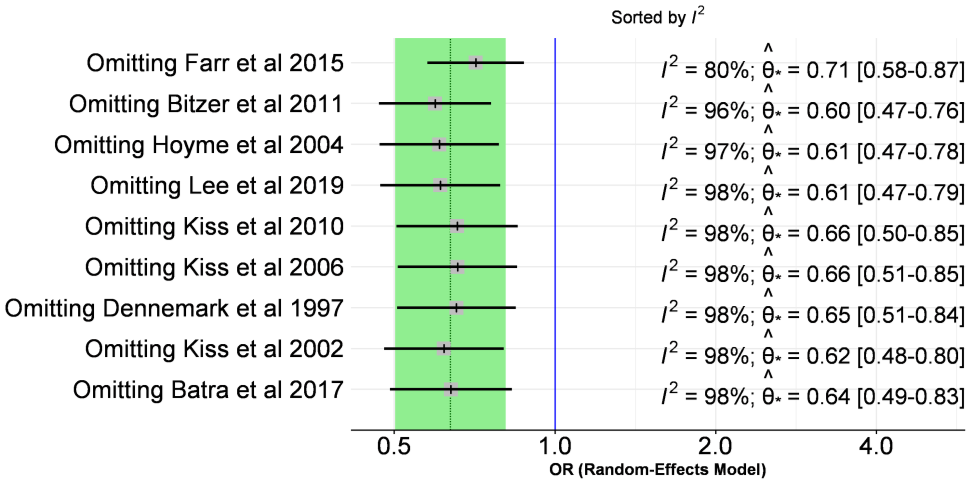
**

**eFigure 15.** Baujat plot for birthweight under 2500g

**
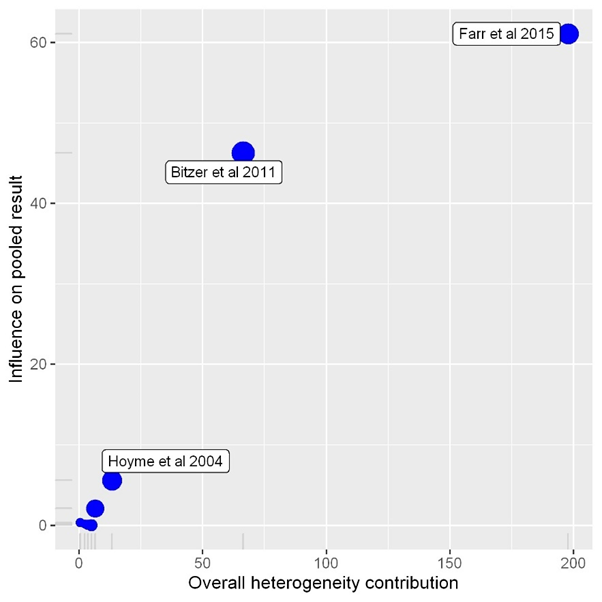
**

**eFigure 16.** Influence diagnostics for birthweight under 2500g

**
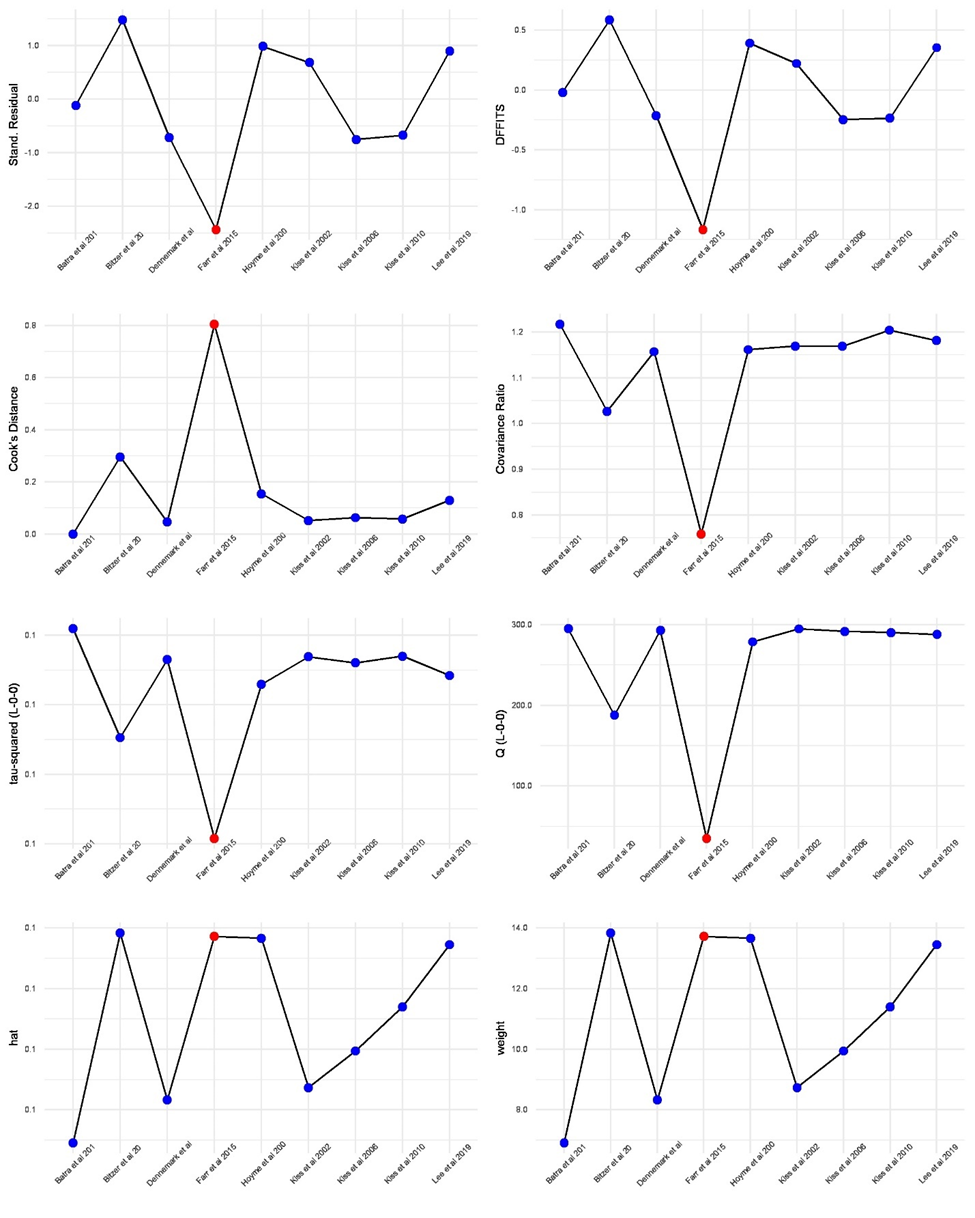
**


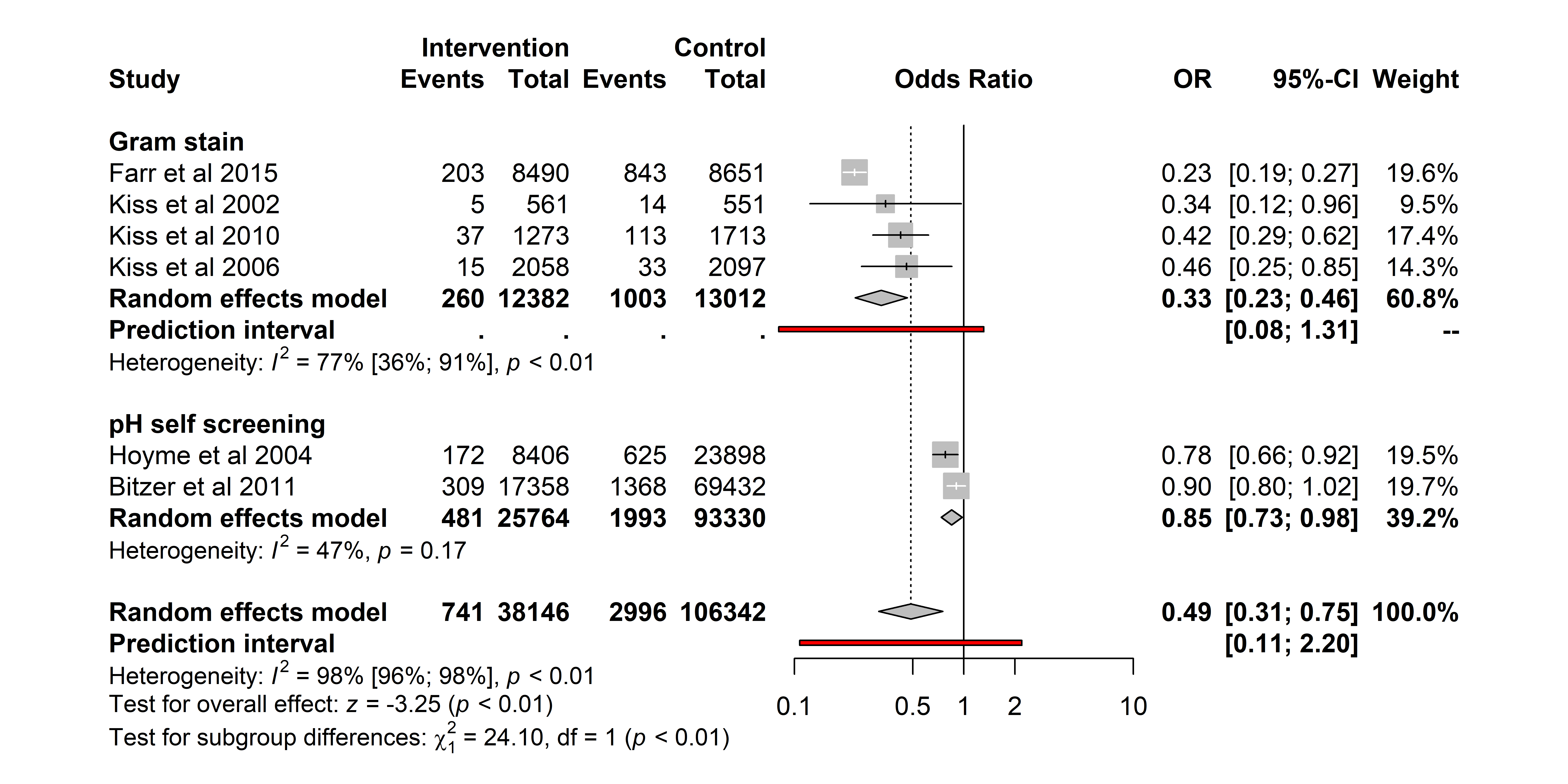
**eFigure 17.** Forest plot representing the odds of birthweight under 2000g

**eFigure 18.** Leave-one-out analysis for birthweight under 2000g

**eFigure 19.** Baujat plot for birthweight under 2000g


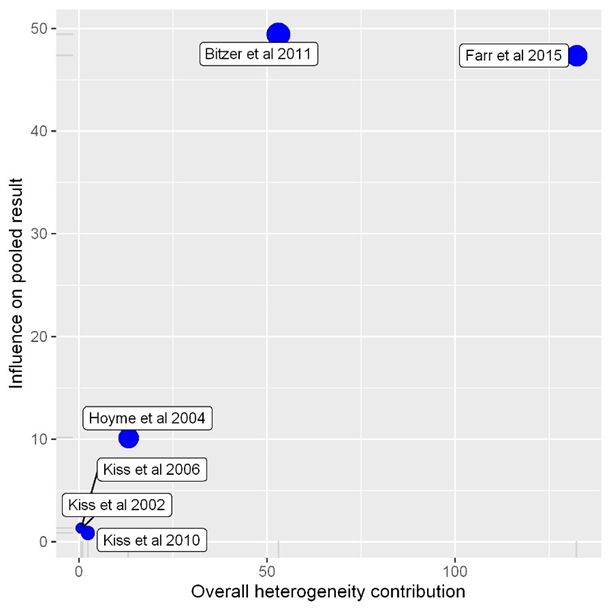


**eFigure 20.** Influence diagnostics for birthweight under 2000g


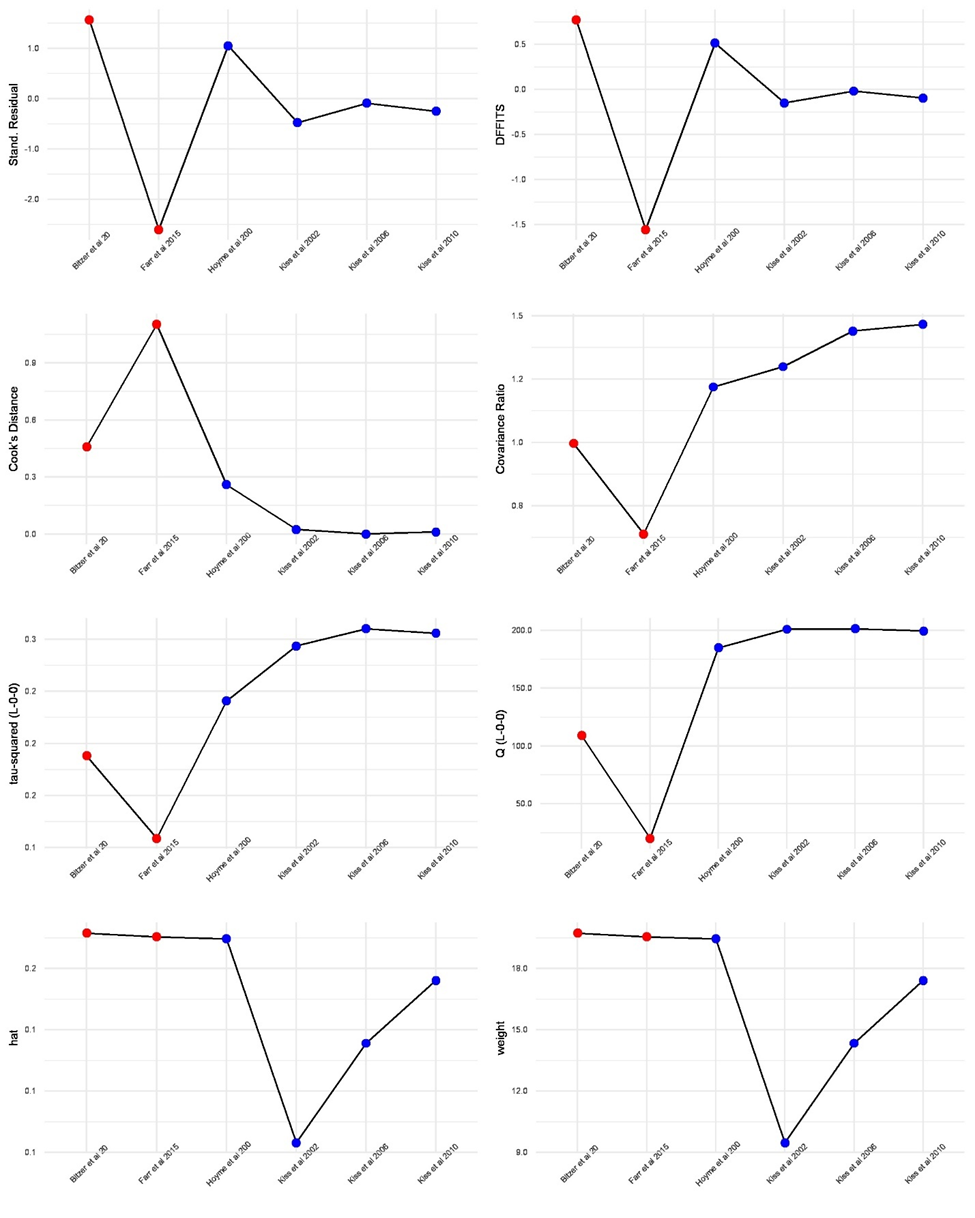


**eFigure 21.** Leave-one-out analysis for birthweight under 1500g

**eFigure 22.** Baujat plot for birthweight under 1500g


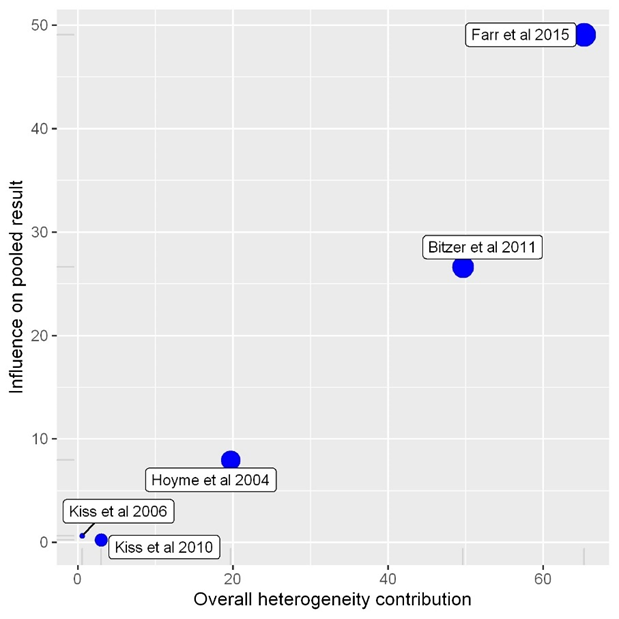


**eFigure 23.** Influence diagnostics for birthweight under 1500g


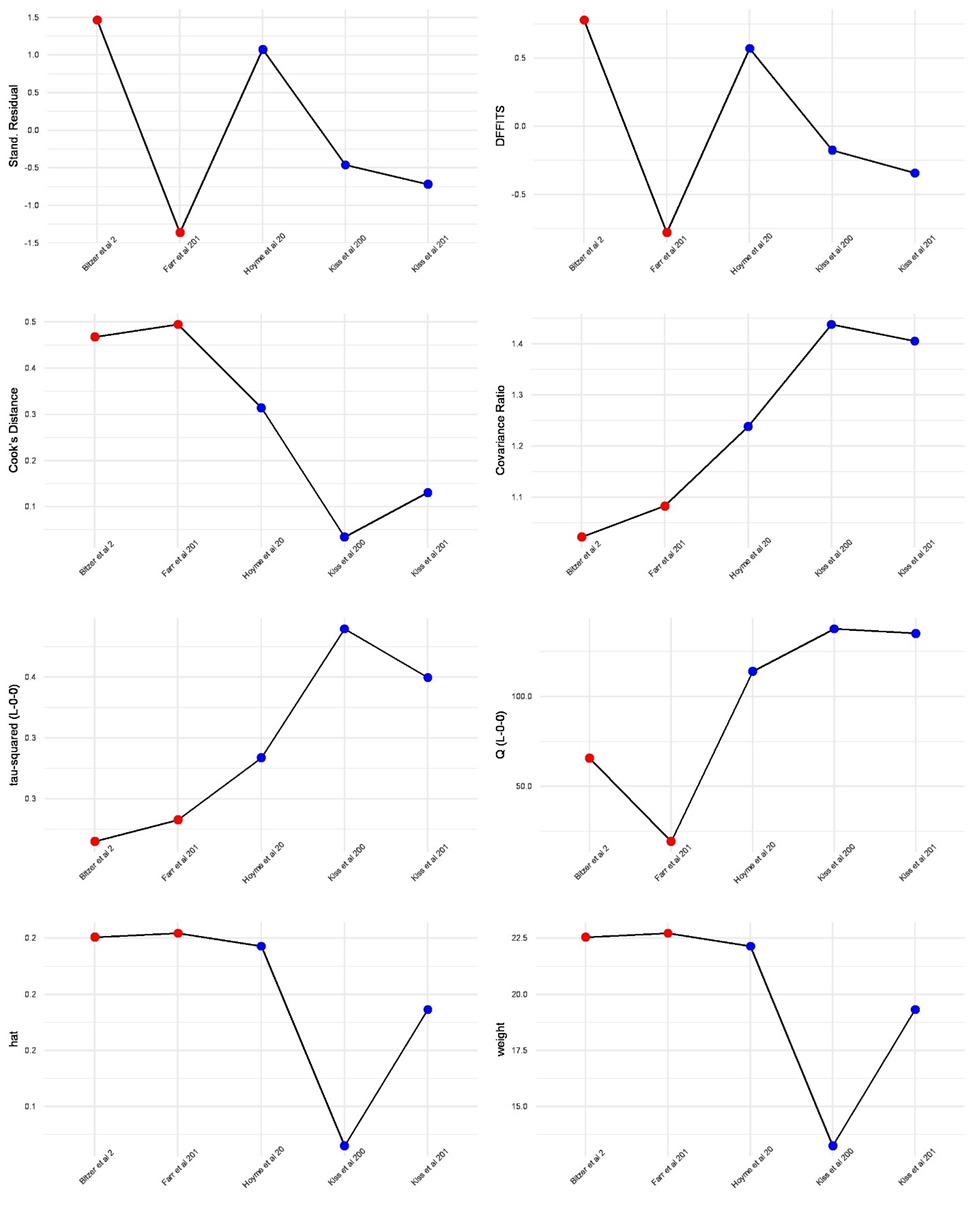


**eFigure 24.** Leave-one-out analysis for birthweight under 1000g

**eFigure 25.** Baujat plot for birthweight under 1000g

**
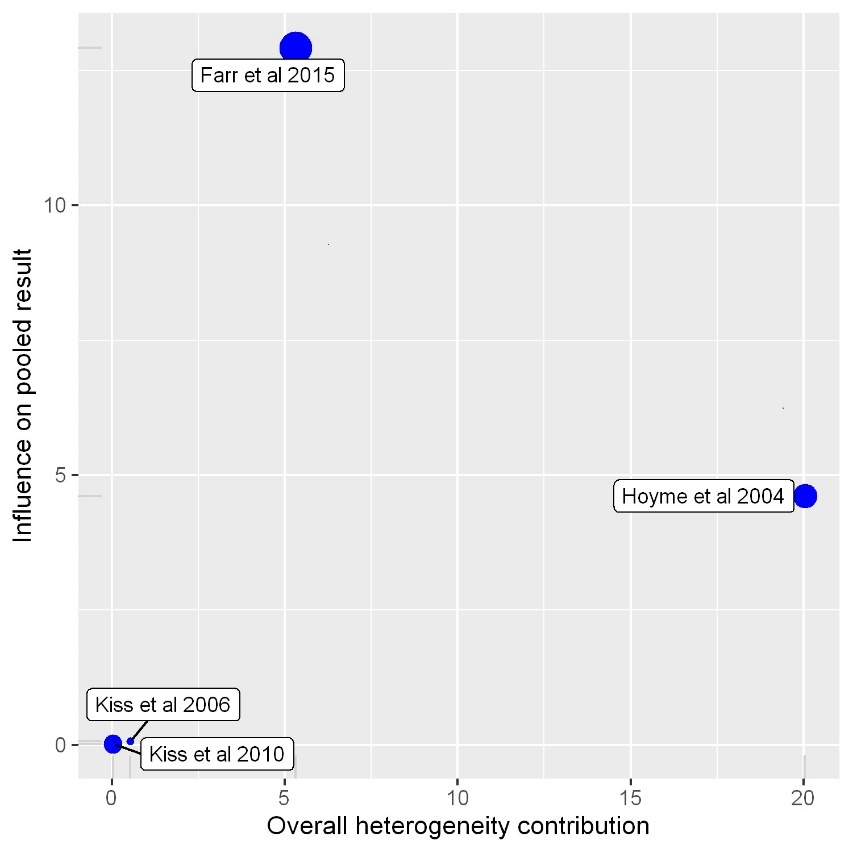
**

**eFigure 26.** Influence diagnostics for birthweight under 1000g

**
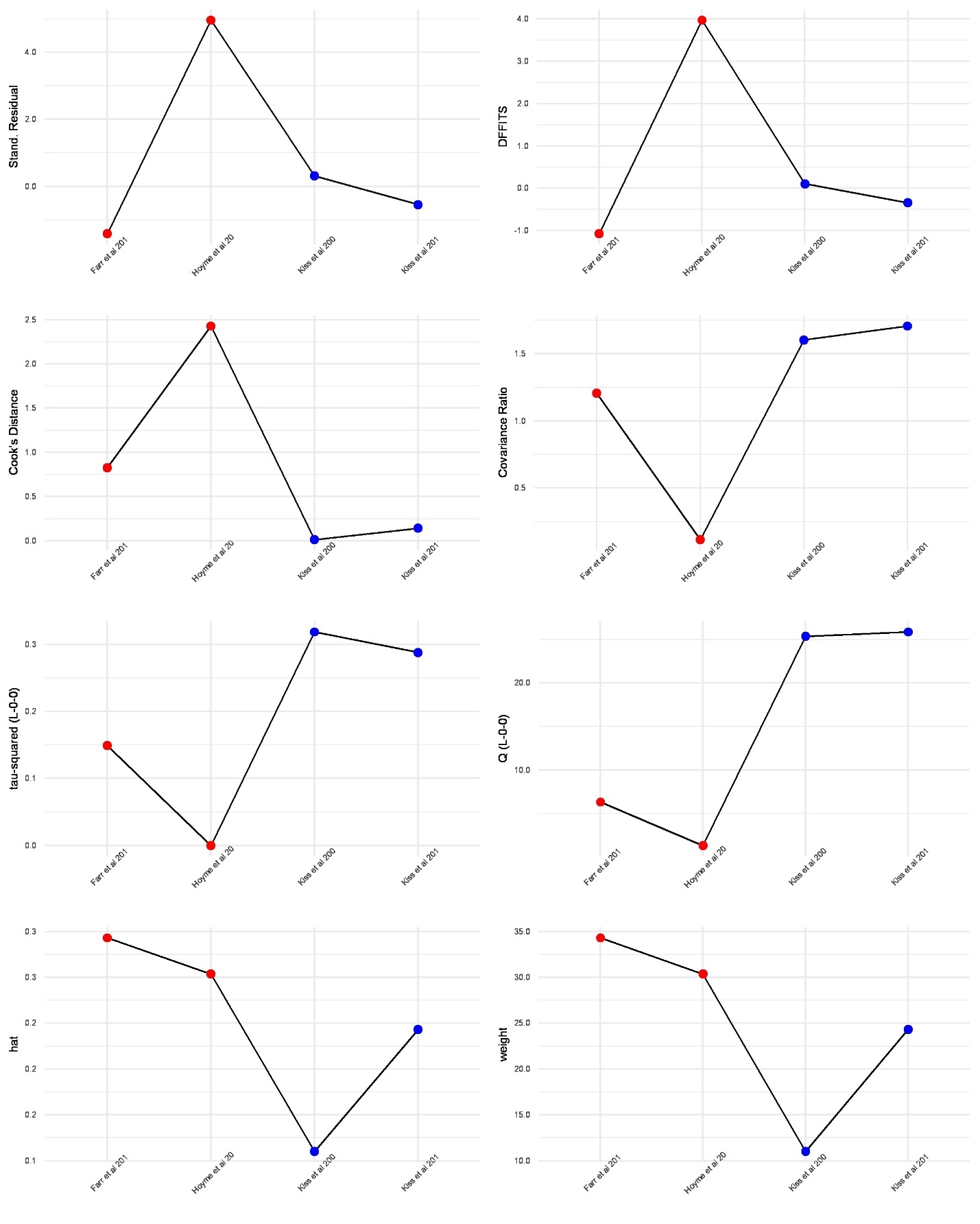
**

**eFigure 27.** Graphical Abstract: Summary of findings. The upper values represent the odds of preterm delivery based on gestation age, while the lower row represents odds of preterm delivery based on birthweight.

**
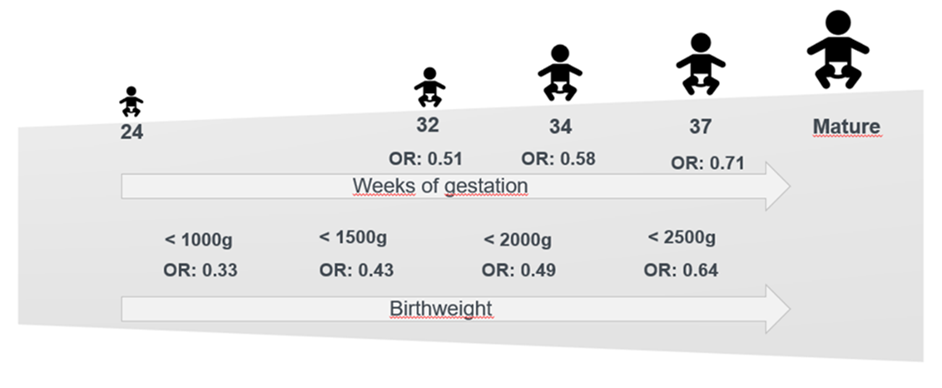
**

**eAppendix 1. Methods**

**Statistical analysis**

Odds ratio (OR) with a 95% confidence interval (CI) was used as an effect size measure. We anticipated considerable between-study heterogeneity in the study population. Therefore, a random-effects model was used to pool effect sizes.

Pooled OR was calculated by the Mantel-Haenszel method. ^14^ Since the Exact Mantel-Haenszel method was used, we did not apply continuity correction to handle zero cell counts (recommended by Cooper, Hedges, and Valentine^15^; J. Sweeting, J. Sutton, and C. Lambert^16^).

For the outcomes where the study number was over 5, a Hartung-Knapp adjustment was used. ^17^ ^18^Below five studies, we do not apply the adjustment.

To estimate the heterogeneity variance measure τ2, the Paule-Mandel method (recommended by Veroniki et al. ^19^) was applied. Additionally, between-study heterogeneity was described using the Cochrane Q test and the Higgins&Thompson's I2. ^20^

In the case of subgroup analysis, we used a mixed-effects model. We assumed that all subgroups share a common τ2 value as we did not anticipate a difference in the between-study heterogeneity in the subgroups, and the study number is relatively small in some subgroups. Therefore, Cochrane’s Q test was used to assess the difference between the subgroups.^21^

Outlier and influence analyses were carried out following the recommendations of Harrer et al. (2021) ^21^ and Viechtbauer and Cheung (2010) ^22^. Publication bias was assessed using Egger's test (at a significance level of 10% as a small study number) with the Harbord method.^23^

**REFERENCE**

1. Lee AC, Mullany LC, Quaiyum M, et al. Effect of population-based antenatal screening and treatment of genitourinary tract infections on birth outcomes in Sylhet, Bangladesh (MIST): a cluster-randomised clinical trial. Article. *Lancet Glob Health*. 2019;7(1):e148-e159. doi:10.1016/S2214-109X(18)30441-8

2. Farr A, Kiss H, Hagmann M, Marschalek J, Husslein P, Petricevic L. Routine Use of an Antenatal Infection Screen-and-Treat Program to Prevent Preterm Birth: Long-Term Experience at a Tertiary Referral Center. Article. *Birth (Berkeley, Calif)*. 2015;42(2):173-180. doi:10.1111/birt.12154

3. Bitzer EM, Schneider A, Wenzlaff P, Hoyme UB, Siegmund-Schultze E. Self-testing of vaginal pH to prevent preterm delivery: a controlled trial. Controlled Clinical Trial; Journal Article; Multicenter Study. *DTSCH ARZTEBL INT*. 2011;108(6):81‐86. doi:10.3238/arztebl.2011.0081

4. Dennemark N, Meyer-Wilmes M, Schlüter R. Screening and treatment of bacterial vaginosis in the early second trimester of pregnancy: A sufficient measure for prevention of preterm deliveries? Article. *Int J STD AIDS*. 1997;8(SUPPL.1):38-40. doi:10.1258/0956462971919435

5. Hoyme UB, Möller U, Saling E. Results and potential consequences of the thuringia prematurity preventional campaign 2000. Article. *GebFra*. 2002;62(3):257-263. doi:10.1055/s-2002-25221

6. Hoyme UB, Saling E. Efficient prematurity prevention is possible by pH-self measurement and immediate therapy of threatening ascending infection. Editorial. *Eur J Obstet Gynecol*. 2004;115(2):148-153. doi:10.1016/j.ejogrb.2004.02.038

7. Sungkar A, Purwosunu Y, Aziz MF, Pratomo H, Sutrisna B, Sekizawa A. Influence of early self-diagnosis and treatment of bacterial vaginosis on preterm birth rate. Journal Article; Multicenter Study; Randomized Controlled Trial; Research Support, Non‐U.S. Gov't. *Int J Gynaecol Obstet*. 2012;117(3):264‐267. doi:10.1016/j.ijgo.2012.01.007

8. Batra A, Bharti R, Sainia P, et al. Evaluation of vaginal pH as a screening tool for bacterial vaginosis and impact of screening and treating for bacterial vaginosis on preterm births. Article. *Indian J Public Health Res Dev*. 2017;8(4):214-219. doi:10.5958/0976-5506.2017.00342.4

9. Kiss H, Petricevic L, Husslein P, Breitenecker G. Infection screening and preterm delivery: The effects of a pathologic vaginal flora on the course of pregnancy. Interim report. Article. *GebFra*. 2002;62(8):762-767. doi:10.1055/s-2002-33714

10. Kiss H, Pichler E, Petricevic L, Husslein P. Cost effectiveness of a screen-and-treat program for asymptomatic vaginal infections in pregnancy: towards a significant reduction in the costs of prematurity. Journal Article; Multicenter Study; Randomized Controlled Trial; Research Support, Non‐U.S. Gov't. *Eur J Obstet Gynecol Reprod Biol*. 2006;127(2):198‐203. doi:10.1016/j.ejogrb.2005.10.017

11. Kiss H, Petricevic L, Martina S, Husslein P. Reducing the rate of preterm birth through a simple antenatal screen-and-treat programme: A retrospective cohort study. Article. *Eur J Obstet Gynecol*. 2010;153(1):38-42. doi:10.1016/j.ejogrb.2010.06.020

12. Gjerdingen D, Fontaine P, Bixby M, Santilli J, Welsh J. The impact of regular vaginal pH screening on the diagnosis of bacterial vaginosis in pregnancy. Clinical Trial; Journal Article; Multicenter Study; Randomized Controlled Trial; Research Support, Non‐U.S. Gov't. *J Fam Pract*. 2000;49(1):39‐43.

13. Nugent RP, Krohn MA, Hillier SL. Reliability of diagnosing bacterial vaginosis is improved by a standardized method of gram stain interpretation. *J Clin Microbiol*. Feb 1991;29(2):297-301. doi:10.1128/jcm.29.2.297-301.1991

14. Mantel N, Haenszel W. Statistical Aspects of the Analysis of Data From Retrospective Studies of Disease. *JNCI*. 1959;22(4):719-748. doi:10.1093/jnci/22.4.719

15. Cooper Harris VHL, C. Valentine Jeffrey. *The Handbook of Research Synthesis and Meta-Analysis, Second Edition*. 2009.

16. J. Sweeting M, J. Sutton A, C. Lambert P. What to add to nothing? Use and avoidance of continuity corrections in meta-analysis of sparse data. <https://doi.org/10.1002/sim.1761>. *Stat Med*. 2004/05/15 2004;23(9):1351-1375. doi:<https://doi.org/10.1002/sim.1761>

17. IntHout J, Ioannidis JPA, Borm GF. The Hartung-Knapp-Sidik-Jonkman method for random effects meta-analysis is straightforward and considerably outperforms the standard DerSimonian-Laird method. *BMC Medical Res Methodol*. 2014/02/18 2014;14(1):25. doi:10.1186/1471-2288-14-25

18. Knapp G, Hartung J. Improved tests for a random effects meta-regression with a single covariate. <https://doi.org/10.1002/sim.1482>. *Stat Med*. 2003/09/15 2003;22(17):2693-2710. doi:<https://doi.org/10.1002/sim.1482>

19. Veroniki AA, Jackson D, Viechtbauer W, et al. Methods to estimate the between-study variance and its uncertainty in meta-analysis. <https://doi.org/10.1002/jrsm.1164>. *JRSM*. 2016/03/01 2016;7(1):55-79. doi:<https://doi.org/10.1002/jrsm.1164>

20. Higgins JP, Thomas J, Chandler J, et al. *Cochrane handbook for systematic reviews of interventions*. John Wiley & Sons; 2019.

21. Mathias Harrer PC, Toshi A. Furukawa, David D. Ebert. *Doing Meta-Analysis with R: A Hands-On Guide*. 2021.

22. Viechtbauer W, Cheung MWL. Outlier and influence diagnostics for meta-analysis. <https://doi.org/10.1002/jrsm.11>. *JRSM*. 2010/04/01 2010;1(2):112-125. doi:<https://doi.org/10.1002/jrsm.11>

23. Harbord RM, Egger M, Sterne JAC. A modified test for small-study effects in meta-analyses of controlled trials with binary endpoints. <https://doi.org/10.1002/sim.2380>. *Stat Med*. 2006/10/30 2006;25(20):3443-3457. doi:<https://doi.org/10.1002/sim.2380>
